# Investigating underlying brain structures and influence of mild and subjective cognitive impairment on dual-task performance in people with Parkinson’s disease

**DOI:** 10.1038/s41598-024-60050-5

**Published:** 2024-04-25

**Authors:** Franziska Albrecht, Hanna Johansson, Urban Ekman, Konstantinos Poulakis, Lucian Bezuidenhout, Joana B. Pereira, Erika Franzén

**Affiliations:** 1 Division of Physiotherapy, Department of Neurobiology, Care Sciences and Society, Karolinska Institutet, Alfred Nobels Allé 23, 141 52, Huddinge, Stockholm, Sweden; 2 Medical Unit Occupational Therapy & Physiotherapy, Women’s Health and Allied Health Professionals Theme, Karolinska University Hospital, Stockholm, Sweden; 3 Stockholm Sjukhem Foundation, Stockholm, Sweden; 4 Division of Neuro, Department of Clinical Neurosciences, Karolinska Institutet, Stockholm, Sweden; 5 Medical Unit Medical Psychology, Women’s Health and Allied Health Professionals Theme, Karolinska University Hospital, Stockholm, Sweden; 6 Division of Clinical Geriatrics, Department of Neurobiology, Care Sciences and Society, Karolinska Institutet, Stockholm, Sweden

**Keywords:** Parkinson’s disease, Mild cognitive impairment, Subjective cognitive impairment, Step-time, Cerebellum, Cerebellum, Parkinson's disease, Cognitive ageing

## Abstract

Cognitive impairment can affect dual-task abilities in Parkinson’s disease (PD), but it remains unclear whether this is also driven by gray matter alterations across different cognitive classifications. Therefore, we investigated associations between dual-task performance during gait and functional mobility and gray matter alterations and explored whether these associations differed according to the degree of cognitive impairment. Participants with PD were classified according to their cognitive function with 22 as mild cognitive impairment (PD-MCI), 14 as subjective cognitive impairment (PD-SCI), and 20 as normal cognition (PD-NC). Multiple regression models associated dual-task absolute and interference values of gait speed, step-time variability, and reaction time, as well as dual-task absolute and difference values for Timed Up and Go (TUG) with PD cognitive classification. We repeated these regressions including the nucleus basalis of Meynert, dorsolateral prefrontal cortex, and hippocampus. We additionally explored whole-brain regressions with dual-task measures to identify dual-task-related regions. There was a trend that cerebellar alterations were associated with worse TUG dual-task in PD-SCI, but also with higher dual-task gait speed and higher dual-task step-time variability in PD-NC. After multiple comparison corrections, no effects of interest were significant. In summary, no clear set of variables associated with dual-task performance was found that distinguished between PD cognitive classifications in our cohort. Promising but non-significant trends, in particular regarding the TUG dual-task, do however warrant further investigation in future large-scale studies.

## Introduction

Parkinson’s disease (PD) is a heterogeneous neurodegenerative disorder characterized by a plethora of motor and non-motor symptoms^1^. Although primarily associated with dopamine deficiency, PD is now also widely recognized to be related to other neurotransmitter deficits. The conceptualization of PD has thus shifted to a multisystem syndrome^2^.

Impaired cognitive function is common even at the time of PD diagnosis, ranging from subjective cognitive impairment (PD-SCI) to mild cognitive impairment (PD-MCI) and PD dementia^3,4^. PD-SCI is characterized as subjective difficulty without demonstrable worsening on cognitive testing. PD-MCI is defined as evidence of cognitive deterioration seen in cognitive tests and self- or informant-reported, but daily living or complex functions may be only minimally impaired^5,6^. Having PD-SCI or PD-MCI is a risk factor for developing dementia^6,7^, and over the course of the disease, approximately 80% of people with PD will develop PD dementia^8^. Compared to only 9% of people with PD with normal cognition (PD-NC), 77% of people with PD-MCI develop dementia within 9 years^9^. Although research on subjective cognitive impairment is sparse, one study showed that 33% of people with PD-SCI convert to dementia within 7.5 years^10^. Imaging studies have also revealed how these prodromal stages of PD dementia are related to brain alterations. Compared to PD-NC, cortical thinning has been found in parahippocampal, frontal, and posterior cortical areas in PD-SCI^11^, whereas reduced connectivity between the precuneus and the bilateral superior parietal lobules has been seen in people with PD-MCI^12–14^.

Similarly to cognitive impairment, gait disturbances may also be present in the early stages of PD^15,16^. In line with PD progression, gait speed tends to decrease, step length shortens, and arm swing amplitude decreases^17^. These deficits become even more prominent during dual-task walking, i.e., walking while simultaneously performing another task, typically a cognitive task. A gradual loss of automaticity, together with impaired executive function, is believed to partly explain dual-task difficulties in people with PD^18^. The ratio by which performance changes from single to dual-task is usually expressed as dual-task interference^19^, and has been suggested as a proxy measure for automaticity and attention^20^. The relationship between cognition and dual-task walking has not yet been investigated in PD-SCI, but studies comparing PD-MCI to PD-NC show that gait is more affected when having to divide attention while walking in PD-MCI^21,22^. Further, it has been shown that dual-task step length could predict a future cognitive decline (attention and executive dysfunction) in PD^23^. The extent to which differences in dual-task performance across cognitive classifications in PD are also accompanied by differences in brain structure remains unknown but could yield insights into the neural underpinnings of dual-task performance. A better understanding of the mechanisms behind dual-task deficits in people with PD can improve the ability to track disease progression and cognitive classification as well as help tailor interventions accordingly. In MCI without PD, dual-task performance, i.e. dual-task gait and cognitive performance, have been shown to be related to various gray matter structures such as the entorhinal cortex^24,25^. To the best of our knowledge, dual-task performance has only been related to gray matter structures in people with PD, without confirmed cognitive impairment^26,27^. Therefore, the aim of this study is to investigate whether cognitive classification and prespecified brain regions-of-interest (ROI) are associated with dual-task performance during gait and functional mobility in people with PD. We aimed to see if those measures and their interaction can differentiate between PD-MCI, PD-SCI, and PD-NC.

We hypothesize that dual-task performance will be most compromised in the PD-MCI group, shown by lower dual-task gait speed, higher dual-task step-time variability, as well as greater dual-task interference on both gait speed and step-time variability, when compared to PD-NC, and to a lower extent when compared to PD-SCI, to be shown in a statistical group effect^22^. Moreover, we hypothesize that dual-task performance will be negatively correlated to gray matter volume in the following manner: PD-MCI > PD-SCI > PD-NC, to be shown in an interaction effect of the group by ROI. According to previous studies in PD and dual-tasking, we expect that the regions showing a negative correlation with dual-task performance will be the hippocampus^28,29^, dorsolateral prefrontal cortex (DLPFC)^30^, and the Nucleus Basalis of Meynert (NBM)^26,27^. In whole-brain regressions, we explore further regions related to dual-tasking performance. In addition, we expect atrophy in areas comprising the hippocampus, insula, putamen, claustrum, and the globus pallidus in whole-brain analyses when comparing PD-MCI with healthy controls (HC) using whole-brain voxel-based morphometry^31^. We do not expect atrophy in the PD-NC vs. HC comparison.

## Results

### Differences in dual-task performance, PD cognitive classifications, and other demographics

In summary, PD-MCI performed worse compared to PD-NC on measures of dynamic balance (Mini-BESTest), functional mobility (TUG single- and dual-task), self-reported depression (HADS), and global cognition (MoCA). PD-MCI also walked with higher step-time variability during dual-task gait, and had slower reaction times during both single- and dual-task compared to PD-NC. PD-SCI had worse values compared with PD-NC in the TUG dual-task and HADS depression scale. PD-MCI had worse values compared with PD-SCI in MoCA, dual-task step-time variability, as well as dual-task gait speed, and dual-task interference on gait speed. There were no differences in age, sex, total intracranial volume (TIV), and MDS-UPDRS-III between PD cognitive classifications. See Table 1, and e-Table 1 for complete list of comparisons.

**Table 1:** Demographics of the participants with Parkinson’s disease (PD) stratified into cognitive classifications.

	NC (*N* = 20)	SCI (*N* = 14)	MCI (*N* = 22)	Total (*N* = 56)	*p* value
*Age, yrs*					0.280^1^
*Mean (CI)*	71.45 (68.77, 74.13)	68.86 (65.52, 72.19)	71.77 (69.12, 74.43)	70.93 (69.36, 72.50)	
*Sex*					0.442^2^
*male*	11 (55.0%)	8 (57.1%)	16 (72.7%)	35 (62.5%)	
*female*	9 (45.0%)	6 (42.9%)	6 (27.3%)	21 (37.5%)	
*Education, yrs*					0.314^1^
*N-Miss*	1	0	0	1	
*Mean (CI)*	15.84 (14.57, 17.11)	15.50 (13.43, 17.57)	14.09 (12.47, 15.72)	15.05 (14.15, 15.96)	
*Hoehn & Yahr*					0.150^1^
*Mean (CI)*	2.15 (1.98, 2.32)	2.21 (1.97, 2.46)	2.41 (2.19, 2.63)	2.27 (2.15, 2.39)	
*LEDD, mg*					0.494^1^
*Mean (CI)*	510.30 (324.85, 695.75)	581.36 (414.92, 747.79)	591.25 (456.58, 725.92)	559.87 (470.84, 648.89)	
*MoCA*					< 0.001^1^
*Mean (CI)*	27.05 (26.28, 27.82)	27.86 (26.75, 28.96)	24.36 (23.44, 25.29)	26.20 (25.55, 26.84)	
*Laterality*					0.913^2^
*right*	12 (60.0%)	7 (50.0%)	10 (45.5%)	29 (51.8%)	
*left*	7 (35.0%)	6 (42.9%)	10 (45.5%)	23 (41.1%)	
*bilateral*	1 (5.0%)	1 (7.1%)	2 (9.1%)	4 (7.1%)	
*MDS-UPDRS III*					0.082^1^
*Mean (CI)*	26.90 (23.06, 30.74)	25.07 (20.31, 29.83)	34.32 (28.26, 40.37)	29.36 (26.34, 32.38)	
*MDS-UPDRS Total*					0.064^1^
*Mean (CI)*	41.05 (35.50, 46.60)	46.93 (38.96, 54.90)	55.68 (46.48, 64.89)	48.27 (43.63, 52.90)	
*Mini-BESTest*					0.009^1^
*Mean (CI)*	22.25 (21.42, 23.08)	21.43 (18.97, 23.89)	19.14 (17.43, 20.84)	20.82 (19.86, 21.79)	
*TUG single-task*					0.018^1^
*Mean (CI)*	9.45 (8.58, 10.32)	10.58 (8.77, 12.39)	11.46 (10.34, 12.59)	10.53 (9.83, 11.22)	
*TUG dual-task*					0.013^1^
*N-Miss*	1	0	0	1	
*Mean (CI)*	13.09 (11.03, 15.16)	16.60 (13.53, 19.67)	16.19 (13.82, 18.57)	15.23 (13.83, 16.62)	
*ABC*					0.076^1^
*Mean (CI)*	85.09 (79.33, 90.86)	81.34 (73.11, 89.57)	73.44 (65.80, 81.08)	79.58 (75.42, 83.73)	
*Walk 12*					0.105^1^
*Mean (CI)*	9.30 (6.38, 12.22)	8.79 (5.74, 11.83)	15.09 (10.82, 19.36)	11.45 (9.30, 13.59)	
*Steps per day*					0.967^1^
*N-Miss*	0	0	3	3	
*Mean (CI)*	5870.53 (4624.44, 7116.62)	6113.34 (4543.05, 7683.63)	6097.88 (4494.50, 7701.26)	6016.17 (5222.88, 6809.46)	
*HADS Anxiety*					0.055^1^
*N-Miss*	0	0	1	1	
*Mean (CI)*	2.75 (1.65, 3.85)	4.64 (2.95, 6.33)	4.57 (3.43, 5.71)	3.93 (3.20, 4.65)	
*HADS Depression*					0.003^1^
*N-Miss*	0	0	1	1	
*Mean (CI)*	1.85 (0.78, 2.92)	3.86 (2.75, 4.96)	4.48 (2.76, 6.20)	3.36 (2.54, 4.19)	
*Nucleaus Basalis of Meynert*					0.064^1^
*Mean (CI)*	0.48 (0.46, 0.49)	0.50 (0.48, 0.51)	0.47 (0.45, 0.49)	0.48 (0.47, 0.49)	
*Hippocampus*					0.877^1^
*Mean (CI)*	0.60 (0.57, 0.63)	0.60 (0.57, 0.64)	0.61 (0.58, 0.63)	0.60 (0.59, 0.62)	
*Dorsolateral prefrontal cortex*					0.838^1^
*Mean (CI)*	0.33 (0.32, 0.34)	0.33 (0.31, 0.34)	0.32 (0.31, 0.34)	0.33 (0.32, 0.33)	
*Cerebellum*					0.018^1^
*Mean (CI)*	0.46 (0.44, 0.47)	0.48 (0.46, 0.50)	0.45 (0.43, 0.46)	0.46 (0.45, 0.47)	
*Single-task gait speed, m/s*					0.506^1^
*Mean (CI)*	1.23 (1.14, 1.33)	1.21 (1.09, 1.34)	1.17 (1.09, 1.26)	1.20 (1.15, 1.26)	
*Dual-task gait speed, m/s*					0.017^1^
*Mean (CI)*	1.19 (1.10, 1.29)	1.24 (1.10, 1.38)	1.06 (0.97, 1.15)	1.15 (1.09, 1.21)	
*DTI gait speed*					0.050^1^
*Mean (CI)*	-3.35 (-7.34, 0.64)	2.28 (-1.26, 5.81)	-8.51 (-14.77, -2.26)	-3.97 (-7.00, -0.94)	
*Single-task steptime variability, ms*					0.074^1^
*Mean (CI)*	20.14 (7.04, 33.25)	14.26 (10.81, 17.70)	17.56 (14.93, 20.18)	17.65 (13.03, 22.28)	
*Dual-task steptime variability, ms*					0.001^1^
*Mean (CI)*	15.89 (13.15, 18.63)	15.54 (12.29, 18.80)	24.82 (20.30, 29.34)	19.31 (16.93, 21.69)	
*DTI steptime variability*					0.048^1^
*Mean (CI)*	9.57 (-10.72, 29.87)	11.35 (0.43, 22.26)	46.46 (21.53, 71.39)	24.51 (11.78, 37.24)	
*Single-task reaction time, s*					0.014^1^
*Mean (CI)*	0.99 (0.92, 1.06)	0.98 (0.90, 1.07)	1.20 (1.08, 1.32)	1.07 (1.01, 1.13)	
*Dual-task reaction time, s*					0.029^1^
*Mean (CI)*	0.93 (0.87, 1.00)	0.95 (0.83, 1.07)	1.09 (1.00, 1.19)	1.00 (0.95, 1.05)	
*DTI reaction time*					0.827^1^
*Mean (CI)*	-5.44 (-8.48, -2.39)	-3.61 (-12.89, 5.67)	-7.46 (-12.68, -2.23)	-5.77 (-8.84, -2.71)	

^1^Kruskal–Wallis-test.

^2^Chi-squared test.

*ABC* Activities-specific balance confidence scale, *CI* Confidence interval, *DTI* Dual-task intereference, *HADS* Hospital anxiety and depression scale, *LEDD* Levodopa equivalent daily dosis, *MDS*-UPDRS Movement disorder society unified parkinson’s disease rating scale, *MiniBEST* Mini balance evaluation systems test, *MoCA* Montreal cognitive assessment, *TUG* Timed up & go test, *yrs* years.

### Associations between dual-task performance and PD cognitive classifications

An overview of all linear regression models can be found in Table 2 and the details of the results are described in the following. The significance level for all analyses was set to *p* < 0.05 and was lowered according to multiple comparisons procedure^32^. We report results that have been significant before correction as “tendencies” and results that were significant after correction as “significances”.

**Table 2:** Summary of the linear regression and ANOVA models.

	Dual-task gait speed	DTI gait speed	Dual-task step-time variability	DTI step-time variability	Dual-task reaction time	DTI reaction time	TUG dual-task	TUG difference
*LR Model (only behavior)*	*	n.s	*	*	*	n.s	*	n.s
*PD-NC*	n.s	n.s	n.s	n.s	n.s	n.s	† PD-MCI	n.s
*PD-SCI*	n.s	n.s	n.s	n.s	n.s	n.s	n.s	n.s
*PD-MCI*	n.s	n.s	†	†	n.s	n.s	n.s	n.s
*Single-task PD-NC*	*		*		*		*	
*Age PD-NC*	n.s	n.s	n.s	n.s	n.s	n.s	†	n.s
*Sex PD-NC*	n.s	n.s	n.s	n.s	n.s	n.s	n.s	n.s
*MDS-UPDRS-III PD-NC*	†	n.s	n.s	n.s	n.s	n.s	†	n.s
PD*single-task	n.s		n.a		n.a		n.a	
PD* MDS-UPDRS-III	n.a	n.a	n.a	n.a	n.a	n.a	* **PD-MCI**	n.a
*ANOVA PD*	n.s	n.s	†	n.s	n.s	n.s	†	n.s
ANOVA PD*single-task	†		n.a		n.a		n.a	
ANOVA PD*MDS-UPDRS-III	n.a	n.a	n.a	n.a	n.a	n.a	†	n.s
*LR Model (brain and behavior)*	*	*Cereb	*	†Cereb	*	n.s	*	n.s
*Brain ROI PD-NC (Cereb, NBM, DLPFC, Hippo)*	n.s	n.s	†DLPFC, Cereb	†Cereb	n.s	n.s	†NBM, Cereb, Hippo	†NBM
*PD-NC*	n.s	†Cereb	*DLPFC, †Cereb	†Cereb	n.s	n.s	n.s	n.s
*PD-SCI*	n.s	n.s	n.s	n.s	n.s	n.s	†Cereb	n.s
*PD-MCI*	n.s	n.s	n.s	n.s	n.s	n.s	†Hippo	n.s
*Single-task PD-NC*	*		*		*		*	
*Age PD-NC*	n.s	n.s	†DLPFC	†Cereb	n.s	n.s	n.s	n.s
*Sex PD-NC*	n.s	n.s	n.s	n.s	n.s	n.s	†	n.s
*MDS-UPDRS-III PD-NC*	n.s	n.s	n.s	n.s	n.s	n.s	†DLPFC, Hippo, Cereb	n.s
PD*Single-task	n.a		n.a		n.a		n.a	
PD* MDS-UPDRS-III	n.a	n.a	n.a	n.a	n.a	n.a	*DLPFC, Hippo, Cereb: PD-MCI	n.s
PD*Age	n.a	n.a	†DLPFC(n.a. for other ROI)	n.a	n.a	n.s	n.a	n.a
PD*ROI	n.s	n.s	n.s	n.s	n.s	n.s	†Hippo, Cereb: PD-SCI	n.s
*ANOVA PD*	n.s	n.s	n.s	n.s	n.s	n.s	†Hippo, Cereb	†Cereb
ANOVA PD*ROI	n.s	n.s	n.s	n.s	n.s	n.s	†Cereb	†Cereb
ANOVA PD*MDS-UPDRS-III	n.a	n.a	n.a	n.a	n.a	n.a	†DLPFC, Hippo, Cereb	n.a

*significant after correction for multiple comparisons.

^†^Trend/ significant before correction for multiple comparisons.

*ANOVA* Analysis of variance of the linear regression model, *Cereb* Cerebellum, *DLPFC* Dorsolateral prefrontal cortex, *DTI* Dual-task interference, *Hippo* Hippocampus, *LR* Linear regression, *MDS-UPDRS-III* Movement disorder society unified parkinson’s disease rating scale, *NBM* Nucleus basalis of meynart, *n.s.* Not significant, *n.a.* Not applicable (likelihood ratio tests were not significant for the interaction term), *PD* Parkinson’s disease, *ROI* Region-of-interest, *TUG* Timed up & go test.

#### Gait speed

Dual-task gait speed was associated with an interaction of PD cognitive classification and single-task gait speed, while sex, MDS-UPDRS-III, and age were included as additive covariates (e-Table 2). This model was significant (F(8,47) = 16.33, *p* < 0.001, R^2^ = 0.74) with single-task gait speed (beta = 0.78, *p* < 0.001) being the only significant variable. In the ANOVA, single-task gait speed was significant and PD cognitive classification by single-task gait speed interaction and MDS-UPDRS-III showed a trend that did not survive the correction for multiple comparisons as a considerable number of models were trained in this study. Dual-task interference was not associated with PD cognitive classification, age, sex, and MDS-UPDRS-III (e-Table 3).

#### Step-time variability

**Figure 1:**
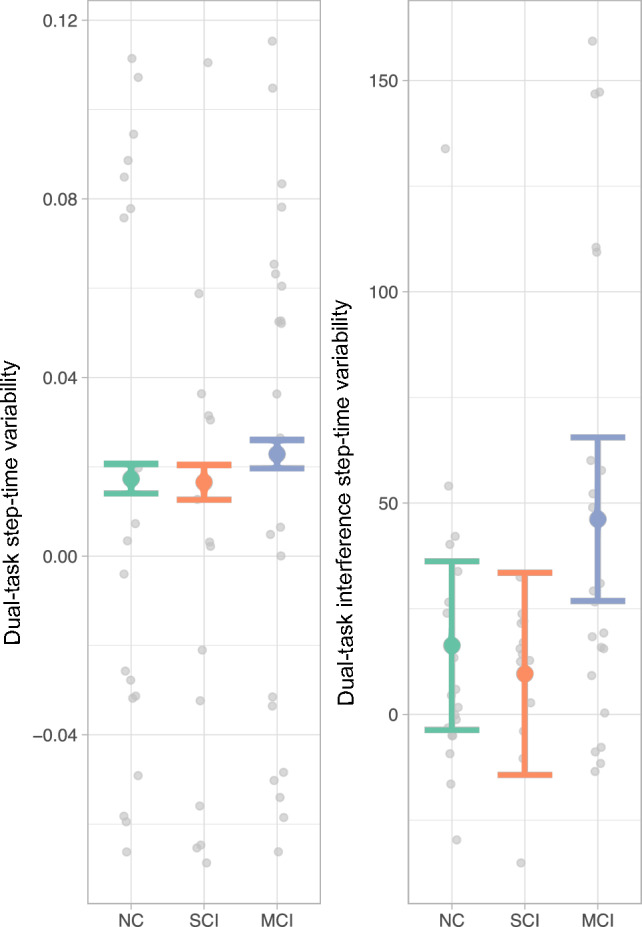
Variable effect plot of dual-tasking variables over Parkinson’s disease cognitive status corrected for effects of age, sex, and MDS-UPDRS-III. The colored dot shows the beta value while the whiskers are the 95% confidence interval. The gray dots display the observed values with the effects of all the control variables accounted for. The raw values were measured as dual-task step-time variability in ms and dual-task interference step-time variability in %. *DTI* = Dual-task interference, *MDS-UPDRS-III* = Movement disorder society unified parkinson’s disease rating scale, *MCI* = Parkinson’s disease with mild cognitive impairment, *NC* = Parkinson’s disease with normal cognition, *SCI* = Parkinson’s disease with subjective cognitive impairment.

The association between dual-task step-time variability and single-task step-time variability, PD cognitive classification, sex, age, and MDS-UPDRS-III as additive covariates was significant (F(6,48) = 7.18, *p* < 0.001, R^2^ = 0.47) (e-Table 4). PD-MCI showed a trend (beta = − 0.0055, *p* = 0.3) and single-task step-time variability (beta = 0.82, *p* = 0.013) was a significant variable (Fig. 1). In the ANOVA, single-task step-time variability was significant and PD cognitive classification showed a trend but was not a significant predictor after multiple comparisons. Dual-task interference on step-time variability was not explained by the regression model (F(5,49) = 2.47, *p* = 0.045, R^2^ = 0.20) although a trend was seen in the PD-MCI variable (beta = 29.9, *p* = 0.4) (Fig. 1, e-Table 5).

#### Timed up & go test

The association of TUG dual-task with PD cognitive classification, TUG single-task, sex, and age, as covariates, and the interaction of the PD cognitive classification by MDS-UPDRS-III was significant (F(6,48) = 7.58, *p* < 0.001, R^2^ = 0.49) (e-Table 8). TUG single-task (beta = 1.09, *p* < 0.001) as well as the interaction of PD cognitive classification with MDS-UPDRS-III (beta = 0.38, *p* = 0.04) were significant variables. PD-MCI, sex, and MDS-UPDRS-III showed a trend to be associated with TUG dual-task. The ANOVA showed that TUG single-task was the only significant variable, while the interaction of PD cognitive classification with MDS-UPDRS-III, sex, and MDS-UPDRS-III showed a trend that did not survive multiple comparisons (e-Table 8). The TUG difference model was not significant (e-Table 9).

### Brain alterations

#### Associations between dual-task performance, PD cognitive classifications, and gray matter structures

An overview of all linear regression models can be found in Table 2 and the details of the results are described in the following.

#### Voxel-wise regression

Our exploratory voxel-wise regression analyses showed that dual-task gait speed correlated with the right cerebellum volume (Family-wise error correction, FWE *p* < 0.05, e-Table 10). This cluster was then extracted as an ROI for the linear models. The regressions with the remaining dual-task variables were not significant.

#### Regions-of-interest

**Figure 2:**
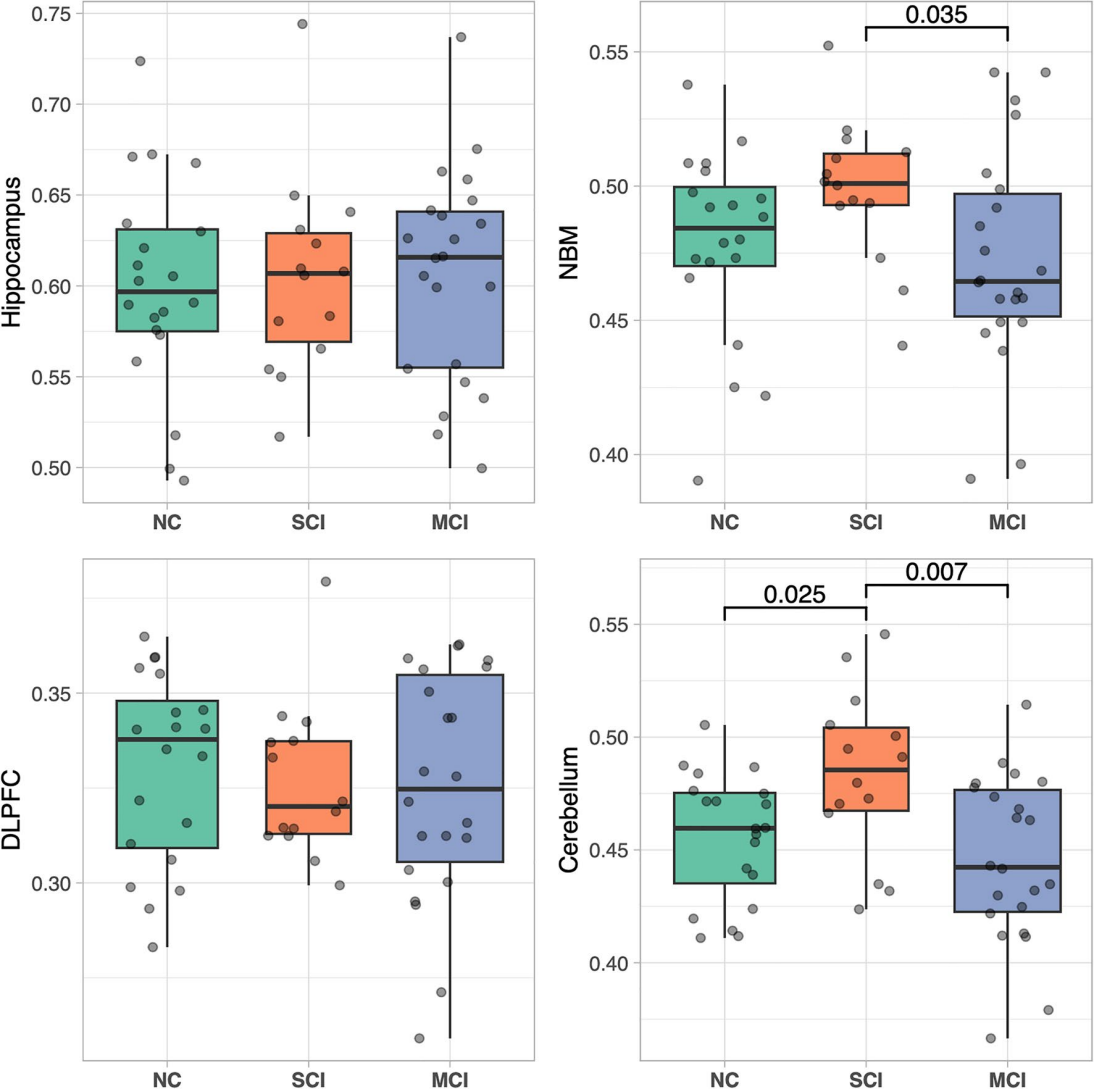
Boxplot of Region-of-Interests (first Eigenvariate) stratified over the Parkinson’s disease cognitive status. *P*-values annotated for significant differences. The gray dots display the observed values. *DLPFC* = Dorsolateral prefrontal cortex, *MCI* = Parkinson’s disease with mild cognitive impairment, *NC* = Parkinson’s disease with normal cognition, *NBM* = Nucleus basalis of meynert, *SCI* = Parkinson’s disease with subjective cognitive impairment.

We found significant differences when comparing the extracted predefined ROIs (i.e., first Eigenvariate) between the PD cognitive classifications and the NBM (PD-SCI > PD-MCI, *p* = 0.04) and the cerebellum (PD-SCI > PD-MCI, *p* = 0.007; PD-SCI > PD-NC, *p* = 0.03), but not for hippocampus or DLPFC (Fig. 2).

#### Gait speed and ROIs

All linear regression models including NBM, hippocampus, DLPFC, or cerebellum as variables were significant and single-task gait speed was always the only significant variable (e-Tables 11, 13, 15, 17).

For dual-task interference on gait speed, the NBM, DLPFC, and hippocampus models were non-significant (e-Tables 12, 14, 16). The cerebellum dual-task interference model was significant (F(8,46) = 2.67, *p* = 0.03, R^2^ = 0.32) and PD-NC, although non-significant, showed a trend (e-Table 18). Regarding the ANOVAs, single-task gait speed was always the only significant variable.

#### Step-time variability and ROIs

The dual-task step-time variability model including NBM was significant (F(9,45) = 5.10, *p*- = 0.002, R^2^ = 0.51), and step-time variability single-task was a significant variable (beta = 0.76, *p* = 0.01) (e-Table 19). The ANOVA underlined that single-task step-time variability was the only significant variable.

The dual-task step-time variability model including DLPFC was significant (F(10,44) = 6.78, *p* < 0.001, R^2^ = 0.61) (e-Table 21, with PD-NC (beta = 0.51, *p* = 0.042), and step-time variability single-task (beta = 0.61, *p* = 0.05) being significant. DLPFC, age, and DLPFC by age interaction showed a trend towards a significant association. In the ANOVA, single-task step-time variability and the interaction age by DLPFC were significant, while DLPFC and age showed only a trend.

The dual-task step-time variability model including the hippocampus was significant (F(9,45) = 4.59, *p* = 0.005, R^2^ = 0.48), with single-task step-time variability being a significant variable (beta = 0.78, *p* = 0.01) (e-Table 23). In the ANOVA, single-task step-time variability was the only significant variable.

The dual-task step-time variability model with the cerebellum as a variable was significant (F(9,45) = 6.52, *p* < 0.001, R^2^ = 0.57) and single-task step-time variability a significant variable (beta = 0.77, *p* < 0.001) (e-Table 25). Albeit non-significant, PD-NC, cerebellum, and age showed a trend towards an association. In the ANOVA, single-task step-time variability was significant and cerebellum showed a trend.

No significant results were found for the dual-task interference on step-time variability including the NBM, DLPFC, and hippocampus (e-Tables 20, 22, 24).

The dual-task interference on step-time variability cerebellum model was significant (F(8,46) = 3.26, *p* = 0.03, R^2^ = 0.36) (e-Table 26). PD-NC, cerebellum, and age showed a trend to be associated with dual-task interference on step-time variability. In the ANOVA, cerebellum and age showed a trend that did not survive multiple comparisons.

#### Timed up & go and ROIs

**Figure 3:**
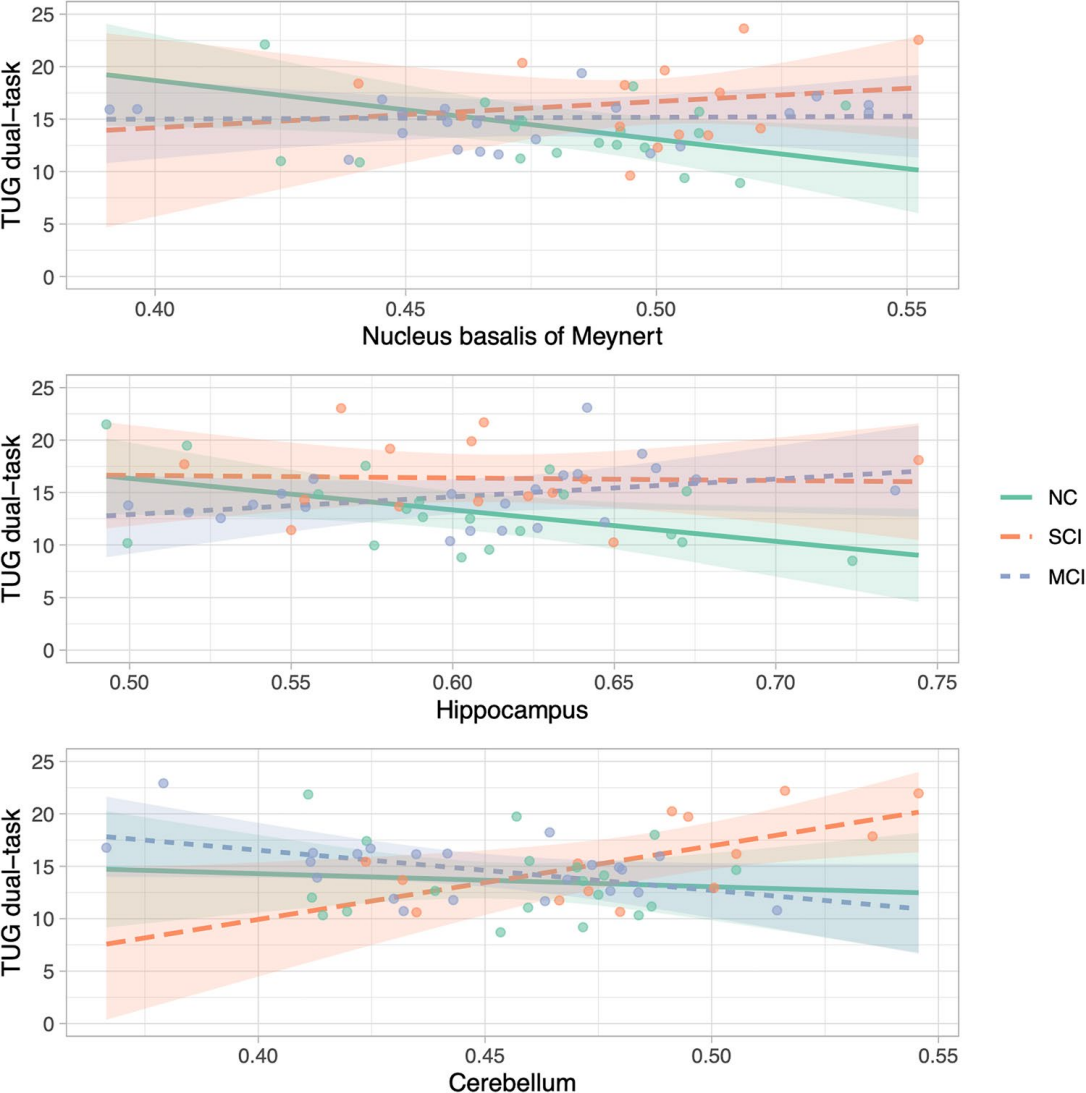
Interaction plot of linear regression models with Region-of-Interest (ROI) as variables. Partial effects plot corrected for age, sex, and MDS-UPDRS-III. The colorful dots display the corrected individual values. The colored shadow displays the 95% confidence interval for the respective group. The dotted lines represent the linear regression lines for each group respectively. ROIs were extracted as the first Eigenvariate and TUG dual-task measured in seconds. *MDS*-*UPDRS*-III = Movement disorder society unified Parkinson’s disease rating scale, *MCI* = Parkinson’s disease with mild cognitive impairment, *NC* = Parkinson’s disease with normal cognition, *SCI* = Parkinson’s disease with subjective cognitive impairment, *TUG* = Timed up & go test.

The model of TUG dual-task as a function of NBM was significant (F(9,45) = 5.95, p < 0.001, R^2^ = 0.54)(e-Table 35), with TUG single-task as a significant variable (beta = 1.14, *p* = 0.001). Although statistically non-significant, the interaction of PD cognitive classification and NBM showed a trend towards an association. In the ANOVA, only TUG single-task was significant, while NBM showed a trend. The DLPFC model for associations with TUG dual-task was significant (F(11,43) = 6.00, *p* < *p* < 0.001, R^2^ = 0.6) (Fig. 3, e-Table 37), and significant variables included TUG single-task (beta = 0.98, *p* = 0.002) and the interaction of PD-MCI and MDS-UPDRS-III (beta = 0.37, *p* = 0.005). The variables sex and MDS-UPDRS-III showed a trend to be significantly associated. The ANOVA also highlighted a trend of significance for the interaction of PD cognitive classification by MDS-UPDRS-III. Further, only TUG single-task was significant and sex and MDS-UPDRS-III showed also a trend. The hippocampus model was significant (F(11,43) = 6.83, *p* < 0.001, R^2^ = 0.64) (Fig. 3, e-Table 39). Significant variables included TUG single-task (beta = 0.97, *p* = 0.002) and the interaction of PD-MCI by MDS-UPDRS-III (beta = 0.42, *p* = 0.04). We found a trend for PD-MCI, hippocampus, sex, MDS-UPDRS-III, and the interaction of PD-MCI by hippocampus, which did not survive multiple comparisons. The ANOVA additionally underlined that PD cognitive classification, the interaction of PD cognitive classification by MDS-UPDRS-III, sex, hippocampus, and MDS-UPDRS-III showed a trend. Only TUG single-task was significant. The cerebellum model was significant (F(9,45) = 6.66, *p* < 0.001, R2 = 0.57) (Fig. 3, e-Table 41). We found a significant effect for TUG single-task (beta = 1.19, *p* = 0.003) and the interaction of PD-MCI with MDS-UPDRS-III (beta = 0.36, *p* = 0.04). The group effect (PD cognitive classification) for PD-SCI, sex, MDS-UPDRS-III, and the interaction of PD-SCI with cerebellum showed a trend. The ANOVA highlighted a trend for the variables PD cognitive classification, PD cognitive classification by cerebellum, PD cognitive classification by MDS-UPDRS-III, sex, and MDS-UPDRS-III. Only TUG single-task was significant.

For TUG difference, the NBM model was not significant. Although NBM exerted a trend in both, the linear regression model as well as the ANOVA, it did not survive multiple comparisons. (e-Table 36). The models including DLPFC, hippocampus, and cerebellum were not significant (e-Tables 38, 40, 42).

#### Voxel-based morphometry

No gray matter differences in the whole brain or when restricting with ROI masks were detected comparing PD-SCI with PD-MCI, PD-MCI with PD-NC, and PD-SCI with PD-NC using an FWE threshold cluster-level *p* < 0.05.

**Figure 4:**
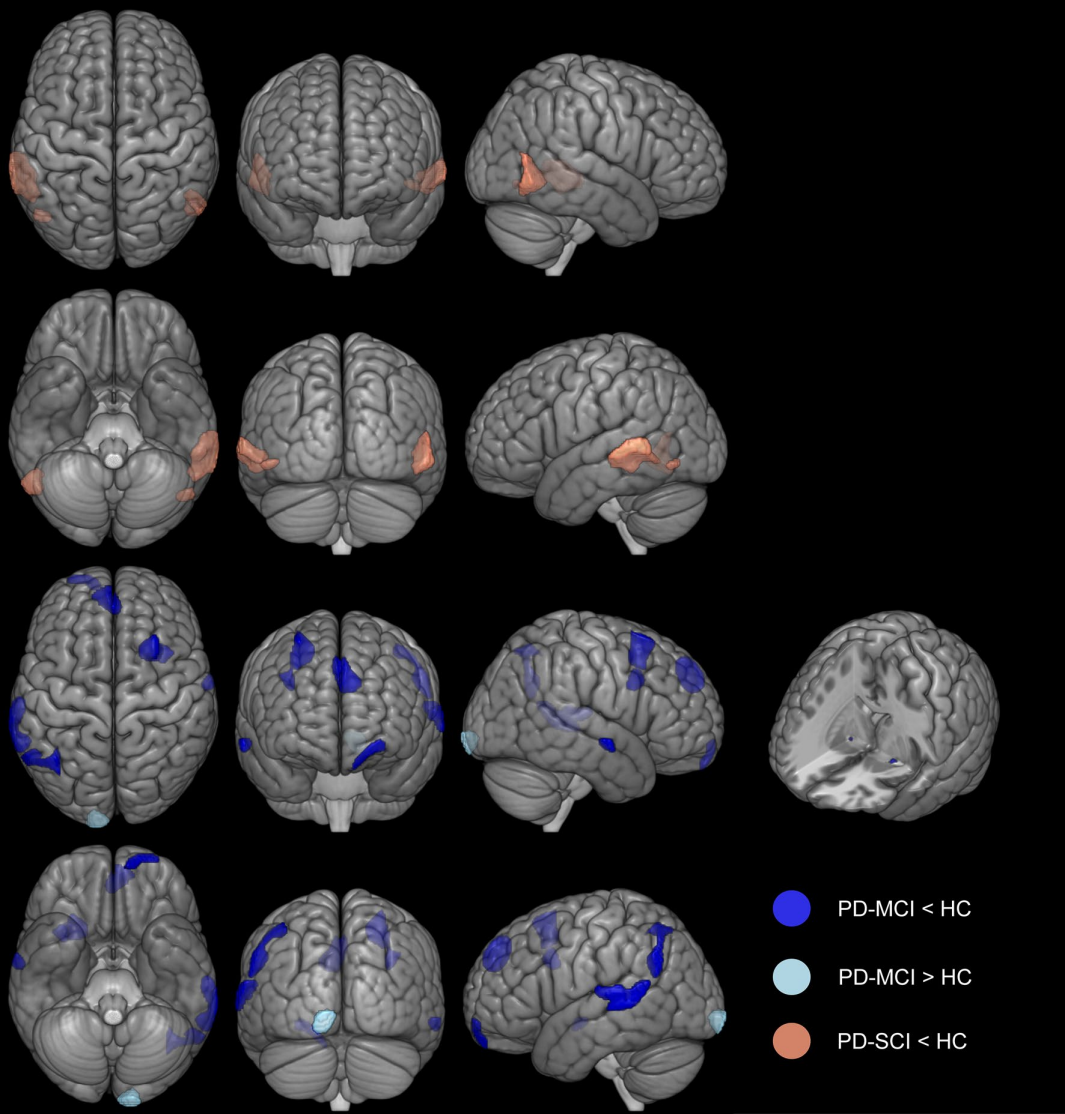
Gray matter volume differences between people with PD and healthy controls (HC). Voxel-based morphometry analyses were corrected with voxel-level *p* < 0.001. Images are shown in neurological convention in the MNI space (the left is on the left). The cut-out shows the NBM Region-of-Interest comparison between PD-MCI and HC. *HC* = Healthy controls, *PD* = Parkinson’s disease, *MCI* = Mild cognitive impairment, *NBM* = Nucleus basalis of meynert, *SCI* = Subjective cognitive impairment.

Using an exploratory threshold (voxel-level *p* < 0.001, k = 100) whole-brain gray matter volume in PD-MCI was lower compared with HC in the right superior/middle frontal gyrus and the right superior/middle temporal gyrus (Fig. 4, e-Table 10). In the left hemisphere, we found lower gray matter volume in the superior frontal, superior/middle temporal, angular/supramarginal gyrus, and frontal pole in PD-MCI compared to controls. Higher gray matter volume in PD-MCI compared with HC was evident in the left occipital pole. PD-SCI showed lower gray matter volume compared with HC in the right hemisphere in the middle/ inferior temporal gyrus and on the left in the middle temporal gyrus and inferior temporal gyrus/ inferior occipital (Fig. 4, e-Table 10).

## Discussion

This study aimed to evaluate whether cognitive classification and its structural brain correlates are associated with dual-task performance in PD. Although a majority of regression models did survive multiple corrections, certain explanatory variables in the models did not. The results suggested non-significant trends for associations between dual-task performance and GM alterations in the hippocampus, DLPFC, and cerebellum. We further hypothesized that dual-task interference on both gait speed and step-time variability would be greater in PD-MCI compared to PD-NC, but this was only partially supported by our analyses. While group comparisons were significant for both dual-task interference on gait speed and step-time variability, our posthoc tests revealed that these were driven by differences between PD-MCI and PD-SCI. Our hypothesis that dual-task performance would be negatively correlated to GM alterations in the following manner PD-MCI > PD-SCI > PD-NC was also only partially supported. Some brain regions showed a trend towards being associated with PD-MCI (hippocampus, DLPFC, and cerebellum) or PD-SCI (cerebellum) only, but no main effect of group for all PD cognitive classifications was found. We expected that the brain regions showing a negative correlation with dual-task performance would be the hippocampus, DLPFC, and NBM. Although tendencies were found between dual-task performance with the hippocampus and DLPFC, the correlations were non-significant and not always negative. The hippocampus showed a trend toward an association between TUG dual-task and PD-MCI in which higher hippocampal alterations were linked to a longer time to complete TUG dual-task, but this was non-significant after multiple corrections. Higher DLPFC alterations were significantly associated with lower step-time variability during dual-task walking, whereas no trends or significant associations were found for NBM.

In addition, we expected atrophy in the left hippocampus, left insula, putamen, claustrum, and right putamen, as well as in the bilateral pallidum in the whole-brain analyses when comparing PD-MCI with HC. Our cohort showed no robust gray matter volume changes when PD groups were compared to each other or to HC. Although we did find atrophy in PD-MCI as compared to HC, it was found only when using a lenient threshold and in other brain regions than the ones we expected. Using this lower threshold, atrophy was also found in the bilateral middle/inferior temporal gyrus in the PD-SCI cohort and frontotemporal atrophy in the PD-MCI cohort. Lastly, we did not expect atrophy in PD-NC compared to HC, and this was supported by our analyses which showed that even with the lower threshold, PD-NC had no atrophy. Among our pre-selected ROIs, only NBM volume differed significantly between the groups (PD-MCI—PD-SCI). In exploratory voxel-wise regressions, we identified the cerebellum as a structure associated with dual-task performance, which also significantly differed in volume between the groups. The finding of an association between dual-task performance and cerebellar alterations and how it differed according to cognitive status is novel and has to our knowledge not been reported previously in people with PD.

Although statistically non-significant in the current study, the cerebellum showed a trend towards an association with dual-task performance and interference. In our sample with mild to moderate PD, a higher cerebellar volume tended to be associated with better dual-task performance in the PD-NC group. Cerebellar involvement in dual-tasking in people with PD has been shown previously^33,34^. Results from an fMRI study indicated that the cerebellum is additionally activated during dual-task in HC compared to people with PD^33^. In that study, the cerebellum was not activated in people with PD during dual-task, but during the pure motor task, a higher activation was evident. Further, people with PD had higher connectivity between the right cerebellum and motor and cognitive networks than HC. The authors concluded that people with PD have limited cerebellar capacity, which might already be overloaded by performing a single task and thus need to integrate motor-cognitive networks to compensate. In general, cerebellar structure and function could be modulated by dopaminergic medication in people with PD. In a recent resting-state fMRI study of 24 people with PD, it has been shown that even a single dosage of levodopa significantly influences the cerebellar connectivity^35^. Here, major connectivity increases between the cerebellum and the motor system regions such as the thalamus, putamen, globus pallidus, and brainstem have been found.

We had hypothesized that NBM structure would be associated with step-time variability but were unable to confirm this. A longitudinal study found NBM volume decreases over time to be predictive of increased step-time variability^26^, but in line with our results, the authors were unable to confirm a cross-sectional relationship.

In contrast to dual-task straight walking, it was instead the TUG dual-task, that showed tendencies to be associated with cognitive status. This is interesting as the TUG is a simple, quick, and widely used clinical test^36^. Compared to straight walking, the TUG also involves transfers (sit-to-stand and stand-to-sit) and turns, which places a higher demand on attentional resources, and especially while simultaneously performing a cognitive task. An additional factor that deserves attention when comparing the two dual-task assessments is the choice of cognitive task. Whereas a serial subtraction task was used during TUG dual-task, an auditory Stroop task was used during straight walking, and it is plausible that this may have contributed to the differing results. Interestingly, when adding ROIs to the analyses we found that the models including NBM, DLPFC, the hippocampus, and the cerebellum all showed tendencies to be associated with the time to complete the TUG dual-task. These results should however be interpreted with caution as these tendencies were non-significant.There was an interaction between hippocampal structure and PD-MCI, and between cerebellar structure and PD-SCI. For PD-MCI the direction of the effect showed that increased TUG dual-task time tended to be associated with greater hippocampal volume. For PD-SCI the effect was observed in the same direction, but here increased time to perform TUG dual-task was associated with higher cerebellar volume. This might be interpreted as a maladaptive compensatory effect on brain structure. Furthermore, as mentioned above, the cerebellum seems to be altered by levodopa treatment in PD^37^. Thus, a volume increase could be related to levodopa intake and may lead to long-term reorganization of the cerebellum that also results in structural changes. The association between increased completion time on the TUG single-task and regional brain volume reductions has been reported previously, but to the best of our knowledge, this is the first study to report an association between TUG dual-task and gray matter volume increases in a PD population^38^.

Except for dual-task step-time variability, models exploring associations between cognitive status and dual-task interference without adding brain ROIs as variables were not significant. The interference that occurs during dual-tasking has been suggested as a proxy measure for automaticity, and previous research has been able to explain small parts of its variance by for example cognition and disease severity^20,22^. Although described extensively in the literature, dual-task interference is however an exploratory variable that lacks validation and cutoff scores in the PD population. This may have consequences for any predictive ability of cognitive status or other behavioral variables, but also for our ability to interpret any significant findings. For this reason, we suggest reporting absolute values of dual-task performance controlled for single-task performance, as a complement to DTI outcomes.

This study has several limitations worth noting. Firstly, we used baseline data from an RCT (the EXPANd trial)^39^ involving highly challenging balance training. Thus, there are potential biases as a result of the secondary nature of the data. People voluntarily signed up for participation, which may have biased our sample towards those more inclined to be physically active. The criteria for the RCT further excluded people with Hoehn &Yahr stages 1 and 4, i.e., both people with mild, unilateral motor symptoms, and people with severe motor symptoms. The exclusion of people in the earlier stages in particular may have limited the number of people classified as PD-SCI, and thereby also our ability to generalise our results to this group. Secondly, this study has no healthy controls to compare dual-task performance to. We acknowledge that the smaller sample size is another limitation with respect to structural MRI and linear regression results interpretation. The low sample size led to very conservative corrections for multiple comparisons, which lowered Type I error We would argue that these hypothesis tests, which were significant before correction for multiple comparisons, can be seen as tendencies and should also be considered, since our variables were carefully selected and we just might miss the statistical power to due to the low sample size. Further, one other potential limitation is that we classify the language and visuospatial domains with the items of MoCA, which is not as sensitive as other tests with better psychometric abilities. Thus, the PD-MCI group could potentially be larger. Since the data is cross-sectional, we cannot make any predictions about dual-task performance in relation to PD cognitive status. Future studies should take into account longitudinal data including repetitive sMRI and cognitive impairment classification. Another limitation might be that we did not correct for multiple comparisons, which is debatable. Since we carefully selected variables based on the literature, we refrained from correction but want to note to interpret the results with caution.

In conclusion, no clear set of variables associated with dual-task performance was found that could distinguish between the different cognitive classifications in our sample with mild to moderate PD. However, the TUG test with an added serial subtraction task seemed to be the most promising dual-task measure, which tended to be associated with cognitive status, and with differences in brain structure. This is promising given the widespread use of the TUG dual-task in the clinic, its simple execution, and low cost. As neurorehabilitation is shifting focus from a one-size-fits-all mindset to a personalized precision rehabilitation, clinicians need to mind the cognitive heterogeneity of PD. We therefore propose future research assess a possible association between the TUG dual-task, cognitive status, and brain alterations in larger samples.

## Methods

### Participants

Data were collected within the framework of a randomized controlled EXPANd trial (EXercise in PArkinson’s disease and Neuroplasticity)^40^. The participants underwent a broad and detailed assessment of balance/gait, overall motor impairment, neuropsychological test battery, and MRI before and after the interventions. The present study investigates a subcohort: baseline data of participants with complete gait, neuropsychological, and MRI assessments. For the EXPANd trial, a total of 95 participants were recruited. We also collected data on 39 HC (age = 70.3 ± 4.9 years, male = 27) that underwent MRI. The main inclusion criteria were mild to moderate disease stage of idiopathic Parkinson’s disease, Hoehn & Yahr 2–3, age ≥ 60 years, and a MoCA score ≥ 21. People with claustrophobia or MRI incompatible implants were excluded. For further details, please see the study protocol^40^.

### Demographic and clinical characteristics

Information on age, years of education, disease duration, and medication intake were collected. Motor function and balance were assessed with the MDS-UPDRS^41^ and Mini-BESTest^42^, respectively. As part of the eligibility screening, MoCA^43^ was used as a measure of global cognition. Self-reported balance and gait ability were captured using the Activities-specific Balance Confidence scale (ABC)^44^ and Walk-12^45^, whereas anxiety and depression were assessed using the HADS^46^.

### Parkinson’s disease cognitive classification

Participants underwent a neuropsychological test battery comprising the following domains: executive function, attention/working memory, episodic memory, and visuospatial functions. For executive function, the Color-Word Interference Test (CWIT) and Verbal Fluency from Delis Kaplan Executive Function System (D-KEFS)^47^ were used. For attention/working memory, digit span from Wechsler Adult Intelligence Scale (WAIS)–fourth edition^48^ and Trail Making Test (TMT), Trials I-IV, from D-KEFS^47^, were used. For episodic memory, Rey Auditory Verbal Learning Test (RAVLT)^49^ and Brief Visuospatial Memory Test-Revised (BVMT-R)^50^ were performed. Finally, for visuospatial functions, the Copy condition from BVMT-R was used.

PD-MCI was calculated according to the Movement Disorder Society task force level II category^5^. Results on two neuropsychological tests within each of the aforementioned domains were compared to normative values. Trial IV from CWIT and trial II from verbal fluency (semantic fluency) were used for the executive function domain, digit span (total score), and trial IV of TMT for attention/working memory domain, delayed recall from both RAVLT and BVMT-R for episodic memory domain and copy condition from BVMT-R and the wire cube subtest from MoCA for the visuospatial domain. For the purpose of the classification, the naming and sentence subtests from MoCA were used in order to add the language domain. Depending on their performance in each of the 10 test measures (based on standard deviation (SD) from normative means), participants were given a score between 1 and 4. Cutoffs for each score were: 1 =  ≤ 1 SD, 2 = 1.01 to 1.49 SD, 3 = 1.50 to 1.99 SD, and 4 =  ≥ 2 SD. If a participant scored 3 or 4 on ≥ 2 tests they were classified as PD-MCI. If a participant scored 1 on all tests or had a maximum of one test scored as 2, 3, or 4, they were classified as PD-NC. Participants who scored 2 on ≥ 2 tests or score 2 on one test and 3 or 4 on one test, were put in an indeterminant group and excluded from further analyses.

To group PD-SCI participants, item 1 from the MDS-UPDRS-I was used as a criterion since it is the most common in the literature^6^. Participants scoring “1: slight” on item 1 (“1: Slight: Impairment appreciated by patient or caregiver with no concrete interference with the patient’s ability to carry out normal activities and social interactions.”), but those who are not classified as PD-MCI were deemed as PD-SCI.

Data from 56 people with PD from the EXPANd trial^40^ were eligible and stratified into 22 PD-MCI, 20 PD-NC, and 14 PD-SCI (Table 1).

### Dual-task data

Single and dual-task gait parameters were measured using an electronic walkway system (GAITRite®, active zone: 8.3 m, CIR Systems, Inc., Havertown, PA, USA). The electronic walkway enables evaluation of a large range of spatiotemporal gait parameters, whereof gait speed and step-time variability were chosen for the present study. Gait speed was selected as it is both commonly reported in research as well as a clinically used gait variable. Step-time variability has previously been suggested as a surrogate marker for gait automaticity and was therefore chosen as an additional gait parameter^51^. To ensure steady-state walking, acceleration, and deceleration, distances of three meters on each side of the GAITRite mat were used. During both single and dual-task conditions, participants walked back and forth on the walkway for a total of six trials. The auditory Stroop task, a cognitive task addressing executive function, was introduced and used as the secondary task during the dual-task gait assessment. The auditory Stroop is a valid and reliable task during dual-task gait assessment in people with PD^52,53^.

Participants were presented with the Swedish words for “high” and “low” in congruent and incongruent high and low tones via wireless headphones (RazerTM ManO’War). They were instructed to respond verbally to the corresponding tone, irrespective of which word was presented, as fast as possible. In order to measure accuracy and reaction times, responses were recorded using Audacity (version 2.1.3) and analyzed using MATLAB (R2020b). Accuracy ratios were calculated for each condition. Reaction times were extracted from the beginning of the stimulus to the beginning of the response, and a mean of all answers, irrespective of whether they were correct or incorrect, was calculated.

The Timed Up & Go Test (commonly abbreviated as TUG, but in the current study as TUG single for ease of interpretation), a test of functional mobility, was also used to assess dual-task performance. During TUG single, participants are timed while they rise from a chair, walk 3 m at their usual speed, turn around, walk back, and sit back down on the chair. In this study, TUG single was further performed as a dual-task, in combination with a serial three subtraction task (TUG dual-task) as described in item 14 of the Mini-BESTest^42^. The difference in completion times (in seconds) between TUG single-task and TUG dual-task was included as a variable comparable to dual-task interference (TUG single-task –TUG dual-task = TUG difference).

### Neuroimaging

Structural MRI (sMRI) was acquired on a 3 T Phillips Ingenia scanner with a 15-channel head coil. The structural 3D T1-weighted sequence was acquired using the following parameters: repetition/echo time (TR/TE) = 6.1/2.8 ms and voxel size of 1 × 1x1 mm^3^.

Data were preprocessed by the standard pipeline of CAT12 (version 12.6) in SPM12 (version 7771), i.e., segmentation and spatial normalization while preserving the amount of gray matter volume. Only images for the whole-brain and dorsolateral prefrontal cortex (DLPFC) analyses were smoothed with a 12 mm kernel at full-width half-maximum (FWHM).

The ROIs were selected based on studies investigating dual-tasking and PD^26,27^, namely the Nucleus Basalis of Meynert (NBM), hippocampus, and DLPFC. ROI masks of the NBM and hippocampus were created using the JuBrain Anatomy Toolbox (v3.0)^54^ and the Shirer atlas was applied for the DLPFC^55^. For the multiple regression analyses, ROI gray matter alterations were extracted as the first Eigenvariate (a summary measure) using SPM12. Since these studies were small cohort studies and of different experimental designs, we also explored the whole brain to find additional structures underlying dual-task performance in PD.

### Statistical analyses

#### Demographic, neuropsychological, and dual-tasking data

Participants were excluded from the analysis if values included in the statistical analysis differed by more than 3 SD, which was the case for three participants (two PD-NC and one PD-MCI).

We compared demographic, neuropsychological, and dual-tasking variables using Kruskal–Wallis- or chi-squared tests. Further, for variables that were significant, we performed post hoc group-wise comparisons with the Nemenyi-Damico-Wolfe-Dunn-test^56^.

Dual-task interference was calculated as the relative change in performance from single to dual-task performance from two gait parameters (gait speed and step-time variability) and two cognitive task outcomes (reaction time and accuracy). The following equation as suggested by Kelly, et al.^19^ was used: *Dual-task interference (%)* = *(Dual-task—Single-task)/Single task* × *100.* For gait speed and accuracy, a higher value indicates better performance, whereas for step-time variability and reaction time, a lower value indicates better performance. The interpretation of dual-task interference for gait speed and accuracy is thereby that a positive value indicates a dual-task benefit and a negative value indicates a dual-task cost. For step-time variability and reaction time, however, the interpretation is that a positive value indicates a dual-task cost and a negative value indicates a dual-task benefit.

#### Associations between dual-task and PD cognitive classifications

**Figure 5:**
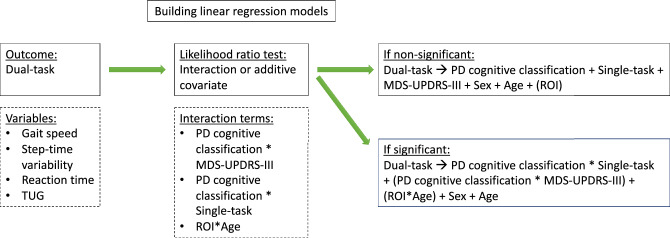
Flow of building the linear regression models. To test if the effect of certain variables on the dual-task measure was different for different PD cognitive classifications (interact with PD cognitive classification), we compared with likelihood ratio tests adding those as interaction terms or additive covariates. For the ROI models, likelihood ratio tests were computed to compare models including the interaction of age and ROI. *MDS*-*UPDRS*-III = Movement disorder society unified parkinson’s disease rating scale, *PD* = Parkinson’s disease, *ROI* = Region-of-interest, *TUG* = Timed up & go test.

We constructed separate linear regression models for each dual-task measure (i.e., gait speed, step-time variability, reaction time, and TUG). We did not construct models for accuracy since the accuracies had a small variance and were deemed as not informative. All models included additive covariates of PD cognitive classifications, sex, age, and motor symptom severity (MDS-UPDRS-III) (Fig. 5). Models of dual-task gait speed, dual-task step-time variability, dual-task reaction time, and TUG dual-task as raw measures (i.e., not calculated dual-task interference) additionally included the respective single-task measure as covariates.

To test if the effect of certain variables on the dual-task measure was different for different PD cognitive statuses (interact with PD cognitive classification), we utilized likelihood ratio tests where interaction or additive terms were added as covariates. These variables were MDS-UPRDS-III and the respective single-task. In case of a significant test, the model with the interaction term was chosen (e.g., PD cognitive classification by single-task). Each linear mixed model was plotted and visually inspected for homoscedasticity and normality of residuals as well as influential observations/outliers using Cook's distance. After building and deciding on the best model, we did ANOVA type III, on the linear regression models to obtain a numerical output of the overall effect of the grouping variable (PD cognitive classification). We reported the significant effects of the grouping variable. All models can be found in the supplement.

The significance level for all analyses was set to *p* < 0.05 and was lowered according to multiple comparisons using the Benjamini–Hochberg procedure^32^. Each comparison using the same group and variable/ term was corrected for the number of tests done on it. We report results that have been significant before correction as “tendencies” and results that were significant after correction as “significances”. Analyses were performed using RStudio (RStudio 2022.07.0 + 548, R version 4.2.1, packages see Supplement)^57^.

#### Associations between dual-task, PD cognitive classifications, and gray matter structures

Whole-brain regressions were computed using the whole group of participants with PD to identify unbiased neural correlates of dual-tasking performance for further investigation as ROI in linear regressions (SPM12). The resulting statistical parametric maps of gray matter differences were corrected for multiple comparisons with an FWE *p* < 0.05 at cluster level and uncorrected *p* < 0.001 voxel level.

For linear regression analyses, we added to the aforementioned models selected ROIs as additional variables (Fig. 5). Likelihood ratio tests were computed to compare models including age and ROI as well as PD cognitive classification and MDS-UDPRS-III as interaction terms and additive covariates. If the likelihood ratio test was significant, the model with the interaction term was chosen. ROI data were corrected for TIV using the residuals of linear regression before entering the linear model ^58,59^. As abovementioned, the constructed linear models were also tested using ANOVA type III. Linear regression analyses employed an alpha level of *p* < 0.05, which was correct according to the abovementioned procedure, and were also computed in RStudio.

We conducted comparisons between all groups (PD-MCI, PD-SCI, PD-NC, HC) to assess structural brain differences in voxel-based morphometry analyses of gray matter volume using separate T-tests in SPM12. We performed these analyses first using a hypothesize-driven ROI approach (ROI as an exclusive mask) and thereafter a whole-brain comparison (voxel-wise comparisons) controlled for TIV and age. We applied an FWE *p* < 0.05 at cluster level and uncorrected *p* < 0.001 voxel level threshold.

### Deviations from preregistration

We preregistered our hypotheses, methods, and analyses for this project at aspredicted.org (aspredicted.org/6jr8u.pdf) and on our OSF page (osf.io/ yw34j/). We deviated from this protocol by applying linear regression instead of ANOVA, because our data violated the assumptions of ANOVA (the gait speed model showed no homogeneity of regression slopes between the covariate and outcome variable – single-task gait speed and dual-task gait speed). Thus, we also did not analyze Hedges *g* but reported standardized beta values for the linear regressions. ANOVA type III were applied on the linear regression models, which we did not preregister. We did not perform analyses on the accuracy values of the cognitive task since there was no meaningful variance in the values and nearly all participants scored maximum values. Thus, the related reaction time analyses (linear regression modles and ANOVA) were placed in the supplement (e-Tables 6, 7, 27–34). Further, we additionally analyzed TUG dual- and single-task because of the clinical value it would add as a quick-assessable test in the clinical routine. Regarding planned exploratory analyses, we did not perform resting-state MRI analyses. Exploratory whole-brain regressions with dual-tasking performance variables were additionally performed.

### Ethics, standard protocol approvals, registrations, and patient consents

The EXPANd trial (clinicaltrials.gov, NCT03213873)^40^ has been approved by the Regional Ethical Review Board in Stockholm (2016/1264–31/4, 2017/1258–32, and 2017/2445–32). Participants received written and oral information about the study and all assessments and provided written informed consent. All research was performed in accordance with relevant guidelines and regulations.

### Supplementary Information

Supplementary Information.

## Data Availability

Data were collected within the framework of the EXPANd trial^40^. The current study was preregistered at aspredicted.org (aspredicted.org/6jr8u.pdf) and is also available on the EXPANd trial OSF page together with the analyses scripts (osf.io/yw34j/). The data generated during this study are not publicly available due to Swedish and EU personal data legislation. Upon a reasonable request to the corresponding author, sharing of the data will be regulated via a data transfer and user agreement with the recipient.
