# Supplementary material for: Investigating underlying brain structures and influence of mild and subjective cognitive impairment on dual-task performance in people with Parkinson’s disease
Source: Sci Rep. 2024 Apr 25;14:9513. doi: 10.1038/s41598-024-60050-5 (PMC11045833; doi:10.1038/s41598-024-60050-5)
Supplement: Supplementary file 1 — Supplementary Information. [file 41598_2024_60050_MOESM1_ESM.pdf]

SUPPLEMENT TO:

Investigating underlying brain structures and influence of mild and subjective cognitive impairment on dual-task performance in people with Parkinson´s disease

1. Table of Contents

1. Methods *3*

R settings 3

2. Results *4*

Associations Between Dual-task Performance and PD Cognitive Classifications 4

Reaction Time 4

Associations Between Dual-task Performance, PD Cognitive Classifications, and Gray Matter Structures 4

Reaction Time and ROIs 4

1. Nemenyi-Damico-Wolfe-Dunn test for the significant tests of the descriptive variables ... 4

2. Linear Regression Model predicting dual-task gait speed by cognitive classification controlled for single-task gait speed (interaction term), sex, age, and MDS-UPDRS III 5

3. Linear Regression Model predicting dual-task interference gait speed by cognitive classification controlled for sex, age, and MDS-UPDRS III 6

4. Linear Regression Model predicting dual-task step-time variability by cognitive classification controlled for single-task step-time variability, sex, age, and MDS-UPDRS III .. 7

5. Linear Regression Model predicting dual-task interference step-time variability by cognitive classification controlled for sex, age, and MDS-UPDRS III 7

6. Linear Regression Model predicting dual-task reaction time by cognitive classification controlled for single-task, sex, age, and MDS-UPDRS III 8

7. Linear Regression Model predicting dual-task interference reaction time by cognitive classification controlled for sex, age, and MDS-UPDRS III 9

8. Linear Regression Model predicting TUG dual-task by cognitive classification controlled for TUG, sex, age, and MDS-UPDRS III 9

9. Linear Regression Model predicting TUG difference by cognitive classification controlled for sex, age, and MDS-UPDRS III 10

10. Local maxima from voxel-based morphometry analysis and voxel-wise regression ... 11

11. Linear Regression Model predicting dual-task gait speed by NBM volume controlled for cognitive classification (interaction term), single-task gait speed, sex, age, and MD-UPDRS III 11

12. Linear Regression Model predicting dual-task interference gait speed by NBM volume controlled for cognitive classification (interaction term), single-task gait speed, sex, age, and MDS-UPDRS III 12

13. Linear Regression Model predicting dual-task gait speed by DLPFC volume controlled for cognitive classification (interaction term), single-task gait speed, sex, age, and MDS-UPDRS III 13

14. Linear Regression Model predicting dual-task interference gait speed by DLPFC volume controlled for cognitive classification (interaction term, sex, age, and MDS-UPDRS III 14

15. Linear Regression Model predicting dual-task gait speed by hippocampus volume controlled for cognitive classification (interaction term), single-task gait speed, sex, age, and MDS-UPDRS III....15

16. Linear Regression Model predicting dual-task interference gait speed by hippocampus volume controlled for cognitive classification (interaction term), sex, age, and MDS-UPDRS III....15

17. Linear Regression Model predicting dual-task gait speed by cerebellum volume controlled for cognitive classification (interaction term), single-task gait speed, sex, age, and MDS-UPDRS III....16

18. Linear Regression Model predicting dual-task interference gait speed by cerebellum volume controlled for cognitive classification (interaction term), single-task gait speed, sex, age, and MDS-UPDRS III....17

19. Linear Regression Model predicting dual-task step-time variability by NBM volumes controlled for cognitive classification (interaction term), total intracranial volume, single-task step-time variability, sex, age, and MDS-UPDRS III....18

20. Linear Regression Model predicting dual-task interference step-time variability by NBM volumes controlled for cognitive classification (interaction term), total intracranial volume, sex, age, and MDS-UPDRS III....19

21. Linear Regression Model predicting dual-task step-time variability by DLPFC volumes controlled for cognitive classification (interaction term), total intracranial volume, single-task step-time variability, sex, age (interaction term), and MDS-UPDRS III....20

22. Linear Regression Model predicting step time variability dual-task by DLPFC volumes controlled for cognitive classification (interaction term), total intracranial volume, step time variability single-task, sex, age, and MDS-UPDRS III....21

23. Linear Regression Model predicting dual-task step-time variability by hippocampus volumes controlled for cognitive classification (interaction term), total intracranial volume, single-task step-time variability, sex, age, and MDS-UPDRS III....21

24. Linear Regression Model predicting dual-task step-time variability by hippocampus volumes controlled for cognitive classification (interaction term), total intracranial volume, sex, age, and MDS-UPDRS III....22

25. Linear Regression Model predicting dual-task step-time variability by cerebellum volumes controlled for cognitive classification (interaction term), total intracranial volume, single-task step-time variability, sex, age, and MDS-UPDRS III....23

26. Linear Regression Model predicting dual-task interference step-time variability by cerebellum volumes controlled for cognitive classification (interaction term), total intracranial volume, sex, age, and MDS-UPDRS II....24

27. Linear Regression Model predicting dual-task reaction time by NBM volumes controlled for cognitive classification (interaction term), single-task reaction time, sex, age, and MDS-UPDRS-III....25

28. Linear Regression Model predicting dual-task interference reaction time by NBM volumes controlled for cognitive classification (interaction term), sex, age, and MDS-UPDRS-III 25

29. Linear Regression Model predicting dual-task reaction time by DLPFC volumes controlled for cognitive classification (interaction term), single-task reaction time, sex, age, and MDS-UPDRS-III....26

30. Linear Regression Model predicting dual-task interference reaction time by DLPFC volumes controlled for cognitive classification (interaction term), sex, age, and MDS-UPDRS-III 27

31. Linear Regression Model predicting dual-task reaction time by hippocampus volumes controlled for cognitive classification (interaction term), single-task reaction time, sex, age, and MDS-UPDRS-III 28

32. Linear Regression Model predicting dual-task interference reaction time by hippocampus volumes controlled for cognitive classification (interaction term), sex, age, and MDS-UPDRS-III 29

33. Linear Regression Model predicting dual-task reaction time by cerebellum volumes controlled for cognitive classification (interaction term), single-task reaction time, sex, age, and MDS-UPDRS-III 29

34. Linear Regression Model predicting dual-task interference reaction time by cerebellum volumes controlled for cognitive classification (interaction term), sex, age, and MDS-UPDRS-III 30

35. Linear Regression Model TUG dual-task by NBM volumes controlled for cognitive classification (interaction term), TUG single-task, sex, age, and MDS-UPDRS-III 31

36. Linear Regression Model TUG difference by NBM volumes controlled for cognitive classification (interaction term), sex, age, and MDS-UPDRS-III 32

37. Linear Regression Model TUG dual-task by DLPFC volumes controlled for cognitive classification (interaction term), TUG single-task, sex, age, and MDS-UPDRS-III (interaction term) 33

38. Linear Regression Model TUG difference by DLPFC volumes controlled for cognitive classification (interaction term), sex, age, and MDS-UPDRS-III 34

39. Linear Regression Model TUG dual-task by hippocampus volumes controlled for cognitive classification (interaction term), TUG single-task, sex, age, and MDS-UPDRS-III (interaction term) 34

40. Linear Regression Model TUG difference by hippocampus controlled for cognitive classification (interaction term), sex, age, and MDS-UPDRS-III 35

41. Linear Regression Model TUG dual-task by cerebellum volumes controlled for cognitive classification (interaction term), TUG single-task, sex, age, and MDS-UPDRS-III (interaction term) 36

42. Linear Regression Model TUG difference by cerebellum volumes controlled for cognitive classification (interaction term), sex, age, and MDS-UPDRS-III 37

1. Methods

R settings

R version 4.2.2 (2022-10-31)
Platform: x86_64-apple-darwin17.0 (64-bit)
Running under: macOS 14.2.1

Matrix products: default
LAPACK: /Library/Frameworks/R.framework/Versions/4.2/Resources/lib/libRlapack.dylib

locale:
[1] en_US.UTF-8/en_US.UTF-8/en_US.UTF-8/C/en_US.UTF-8/en_US.UTF-8

attached base packages:
[1] stats graphics grDevices utils datasets methods base

other attached packages:
[1] ggstance_0.3.6 pgirmess_2.0.0 terra_1.7-18 sjlabelled_1.2.0 sjmisc_2.8.9
[6] sjPlot_2.8.12 ggsignif_0.6.4 patchwork_1.1.2 RColorBrewer_1.1-3 broom_1.0.5

```
[11] ppcor_1.1        lmtest_0.9-40     zoo_1.8-12        huxtable_5.5.2     interactions_1.1.5
[16] jtools_2.2.1     multcomp_1.4-22   TH.data_1.1-1     MASS_7.3-58.1      survival_3.4-0
[21] mvtnorm_1.1-3    car_3.1-2         carData_3.0-5     ggpubr_0.6.0       openxlsx_4.2.5.2
[26] arsenal_3.6.3    lubridate_1.9.2   forcats_1.0.0     stringr_1.5.1      dplyr_1.1.4
[31] purrr_1.0.2      readr_2.1.4       tidyr_1.3.0       tibble_3.2.1       ggplot2_3.4.4
[36] tidyverse_2.0.0  here_1.0.1        knitr_1.45

loaded via a namespace (and not attached):
 [1] minqa_1.2.6       colorspace_2.1-0  deldir_1.0-6      ellipsis_0.3.2    class_7.3-20
 [6] rprojroot_2.0.4   estimability_1.4.1 rstudioapi_0.15.0 proxy_0.4-27     fansi_1.0.6
[11] codetools_0.2-18  splines_4.2.2     nloptr_2.0.3      ggeffects_1.2.0   compiler_4.2.2
[16] sjstats_0.18.2    emmeans_1.8.5     backports_1.4.1   fastmap_1.1.1     assertthat_0.2.1
[21] Matrix_1.6-4      cli_3.6.2         s2_1.1.2          htmltools_0.5.7   tools_4.2.2
[26] gtable_0.3.4      glue_1.6.2        wk_0.7.1          Rcpp_1.0.11       vctrs_0.6.5
[31] spdep_1.2-8       nlme_3.1-160      insight_0.19.0    xfun_0.41         lme4_1.1-35.1
[36] timechange_0.2.0  lifecycle_1.0.4   rstatix_0.7.2     scales_1.3.0      hms_1.1.2
[41] sandwich_3.0-2    yaml_2.3.8        pander_0.6.5      stringi_1.8.3     bayestestR_0.13.0
[46] e1071_1.7-13      boot_1.3-28       zip_2.2.2         spData_2.2.2      rlang_1.1.2
[51] pkgconfig_2.0.3   evaluate_0.23     lattice_0.20-45   sf_1.0-9          tidyselect_1.2.0
[56] magrittr_2.0.3    R6_2.5.1          generics_0.1.3    DBI_1.1.3         pillar_1.9.0
[61] withr_2.5.2       units_0.8-1       datawizard_0.6.5  abind_1.4-5       sp_1.6-0
[66] performance_0.10.2 modelr_0.1.10    crayon_1.5.2      KernSmooth_2.23-20 utf8_1.2.4
[71] rmarkdown_2.25    tzdb_0.3.0        grid_4.2.2        digest_0.6.33     classInt_0.4-9
[76] xtable_1.8-4      munsell_0.5.0
```

2. Results

Associations Between Dual-task Performance and PD Cognitive Classifications

Reaction Time

The model of dual-task reaction time including PD cognitive classification, single-task reaction time, sex, age, and MDS-UPDRS-III was significant ($F(6,49)=15.31$, $p<0.001$, $R^2=0.65$) (e-Table 6). Only single-task reaction time was a significant variable (beta=0.66, p=0.001).

The model of dual-task interference reaction time was not significant (e-Table 7).

Associations Between Dual-task Performance, PD Cognitive Classifications, and Gray Matter Structures

Reaction Time and ROIs

The dual-task reaction time linear regression models including the NBM, DLPFC, or hippocampus or cerebellum as variables were significant and none only single-task reaction time was the significant in all models (e-Table 27, 29, 31).

For dual-task interference on reaction time, the NBM, DLPFC, hippocampus, and cerebellum models and covariates were non-significant (e-Tables 28, 30, 32, 34).

1. Nemenyi-Damico-Wolfe-Dunn test for the significant tests of the descriptive variables

	Observed difference	*Critical difference*	*Significant difference*
MiniBEST 1-2	14.4818182	12.0631189	TRUE
MiniBEST 1-3	2.44285714	13.6057267	FALSE
MiniBEST 2-3	12.038961	13.3486453	FALSE
TUG single-task 1-2	14.2363636	12.0631189	TRUE

TUG single-task 1-3	7.02857143	13.6057267	FALSE
TUG single-task 2-3	7.20779221	13.3486453	FALSE
TUG dual-task 1-2	13.1722488	12.0118252	TRUE
TUG dual-task 1-3	13.7274436	13.508943	TRUE
TUG dual-task 2-3	0.55519481	13.1123674	FALSE
HADS depression 1-2	14.7928571	11.9831915	TRUE
HADS depression 1-3	15.7214286	13.3648983	TRUE
HADS depression 2-3	0.92857143	13.2332213	FALSE
MoCA 1-2	17.3022727	12.0631189	TRUE
MoCA 1-3	5.88928571	13.6057267	FALSE
MoCA 2-3	23.1915584	13.3486453	TRUE
Gait speed dual-task 1-2	10.8954545	12.0631189	FALSE
Gait speed dual-task 1-3	3.72142857	13.6057267	FALSE
Gait speed dual-task 2-3	14.6168831	13.3486453	TRUE
DTI gait speed 1-2	5.07727273	12.0631189	FALSE
DTI gait speed 1-3	8.57857143	13.6057267	FALSE
DTI gait speed 2-3	13.6558442	13.3486453	TRUE
DTI step-time variability 1-2	11.8954545	12.0631189	FALSE
DTI step-time variability 1-3	2.70714286	13.6057267	FALSE
DTI step-time variability 2-3	9.18831169	13.3486453	FALSE
Dual-task step-time variability 1-2	16.0181818	12.0631189	TRUE
Dual-task step-time variability 1-3	0.37142857	13.6057267	FALSE
Dual-task step-time variability 2-3	16.3896104	13.3486453	TRUE
Single-task reaction time 1-2	13.1590909	12.0631189	TRUE
Single-task reaction time 1-3	0.32142857	13.6057267	FALSE
Single-task reaction time 2-3	12.8376623	13.3486453	FALSE
Dual-task reaction time 1-2	12.2318182	12.0631189	TRUE
Dual-task reaction time 1-3	0.97857143	13.6057267	FALSE
Dual-task reaction time 2-3	11.2532468	13.3486453	FALSE
Cerebellum 1-2	12.2285714	13.60573	FALSE
Cerebellum 1-3	3.2000000	12.06312	FALSE
Cerebellum 2-3	15.4285714	13.34865	TRUE

Observed difference are the differences between all groups calculated separately. Critical difference: calculated values for each comparison that needs to be reached to be significant. Abbreviations: 1= PD-NC, 2=PD-MCI, 3=PD-SCI, DTI= dual-task interference, HADS= Hamilton Anxiety and Depression scale, MoCA= Montreal Cognitive Assessment, MiniBEST= Mini Balance Evaluation Systems Test, TUG= Timed up & go test.

2. Linear Regression Model predicting dual-task gait speed by cognitive classification controlled for single-task gait speed (interaction term), sex, age, and MDS-UPDRS III

	**Dual-task gait speed**				
Predictors	*Beta*	*CI (95%)*	*t-stats*	*p*	*p corr.*
Intercept (NC)	0.36	-0.37 – 1.09	1.00	0.32	
MCI	0.27	-0.26 – 0.80	1.03	0.31	

SCI	-0.39	-1.01 – 0.22	-1.29	0.20	
Single-task gait speed	0.78	0.44 – 1.12	4.64	**<0.001**	**0.00022**
Sex	-0.02	-0.09 – 0.06	-0.40	0.69	
MDS-UPDRS III	-0.004	-0.01 – -0.00	-2.24	**0.03**	**0.2**
Age	-0.00	-0.01 – 0.01	-0.04	0.97	
MCI* Single-task gait speed	-0.27	-0.70 – 0.15	-1.29	0.20	
SCI* Single-task gait speed	0.37	-0.12 – 0.87	1.51	0.14	
Observations	56				
R^2 / R^2adjusted	0.735 / 0.690				
F (8,47) = 16.33, p =3.103e-11, p corr. = 6.2e-10					

ANOVA table

	SS	*df*	*F*	*p*	*p corr.*
Intercept (NC)	0.015	1	0.991	0.325	
PD cognitive classification	0.075	2	2.478	0.095	
Single-task gait speed	0.328	1	21.549	**0.000**	**0.0002**
Sex	0.002	1	0.161	0.690	
MDS-UPDRS-III	0.076	1	5.006	**0.030**	0.2
Age	0.000	1	0.002	0.966	
PD cognitive classification*Single-task gait speed	0.109	2	3.584	**0.036**	1
Residuals	0.714	47			

3. Linear Regression Model predicting dual-task interference gait speed by cognitive classification controlled for sex, age, and MDS-UPDRS III

	**Dual-task interference gait speed**			
Predictors	*Beta*	*CI (95%)*	*t-stats*	*p*
Intercept (NC)	-6.70	-44.71 – 31.31	-0.35	0.72
MCI	-4.30	-11.50 – 2.90	-1.20	0.24
SCI	5.64	-2.12 – 13.40	1.46	0.15
Sex	-0.16	-6.35 – 6.02	-0.05	0.96
MDS-UPDRS III	-0.12	-0.41 – 0.16	-0.88	0.38
Age	0.09	-0.42 – 0.61	0.37	0.72
Observations	56			
R^2 / R^2 adjusted	0.159 / 0.074			
F(5,50) = 1.88, p = 0.11				

ANOVA table

	SS	*df*	*F*	*p*
Intercept (NC)	14.867	1	0.125	0.725
PD cognitive classification	707.267	2	2.978	0.060
Sex	0.335	1	0.003	0.958
MDS-UPDRS-III	91.896	1	0.774	0.383
Age	15.983	1	0.135	0.715
Residuals	5938.120	50		

4. Linear Regression Model predicting dual-task step-time variability by cognitive classification controlled for single-task step-time variability, sex, age, and MDS-UPDRS III

	**Dual-task step time variability**				
Predictors	*Beta*	*CI (95%)*	*t-stats*	*p*	*p corr.*
Intercept (NC)	0.01	-0.01 – 0.04	1.08	0.29	
SCI	-0.00	-0.01 – 0.00	-0.33	0.75	
MCI	0.01	0.00 – 0.01	2.33	**0.02**	0.3
Single-task step-time	0.82	0.39 – 1.25	3.83	**<0.001**	**0.013**
MDS-UPDRS III	0.00	-0.00 – 0.00	1.17	0.25	
Sex	0.00	-0.00 – 0.01	0.63	0.53	
Age	-0.00	-0.00 – 0.00	-0.94	0.35	
Observations	55				
R^2 / R^2adjusted	0.473 / 0.407				

$F(6,48) = 7.18$, $p = 1.678e\text{-}05$, p corr. = 3.9 e-05

ANOVA table

	SS	***df***	***F***	***p***	***p corr.***
Intercept (NC)	0.000	1	1.156	0.288	
PD cognitive classification	0.000	2	3.825	**0.029**	0.2
Single-task step-time	0.001	1	14.681	**0.000**	**0.02**
MDS-UPDRS-III	0.000	1	1.358	0.250	
Sex	0.000	1	0.394	0.533	
Age	0.000	1	0.877	0.354	
Residuals	0.002	48			

5. Linear Regression Model predicting dual-task interference step-time variability by cognitive classification controlled for sex, age, and MDS-UPDRS III

	**Step-time variability dual-task interference**				
Predictors	*Beta*	*CI (95%)*	*t-stats*	*p*	*p corr.*

Intercept (NC)	106.74	-42.73 – 256.22	1.44	0.16	
SCI	-6.69	-37.45 – 24.08	-0.44	0.66	
MCI	29.90	1.48 – 58.31	2.11	**0.04**	**0.4**
MDS-UPDRS III	0.54	-0.57 – 1.64	0.97	0.33	
Sex	12.03	-12.53 – 36.60	0.98	0.33	
Age	-1.56	-3.59 – 0.47	-1.54	0.13	
Observations	55				
R^2 / R^2adjusted	0.201 / 0.120				
F(5,49) = 2.47, p = 0.0453, p corr. = 0.08					

ANOVA table

	SS	*df*	*F*	*p*
Intercept (NC)	14.867	1	0.125	0.725
PD cognitive classification	707.267	2	2.978	0.060
Sex	0.335	1	0.003	0.958
MDS-UPDRS-III	91.896	1	0.774	0.383
Age	15.983	1	0.135	0.715
Residuals	5938.120	50		

6. Linear Regression Model predicting dual-task reaction time by cognitive classification controlled for single-task, sex, age, and MDS-UPDRS III

	Dual-task reaction time				
Predictors	*Beta*	*CI (95%)*	*t-stats*	*p*	*p corr.*
Intercept (NC)	0.13	-0.39 – 0.64	0.50	0.62	
MCI	0.00	-0.09 – 0.09	0.06	0.95	
SCI	0.03	-0.06 – 0.12	0.60	0.55	
Single-task reaction time	0.66	0.47 – 0.84	7.11	**<0.001**	**1.78^{e-07}**
Sex	-0.02	-0.10 – 0.05	-0.62	0.54	
MDS-UPDRS III	0.00	-0.00 – 0.01	0.51	0.61	
Age	0.00	-0.00 – 0.01	1.14	0.26	
Observations	56				
R^2 / R^2 adjusted	0.652 / 0.610				
F(6,49) = 15.31, p = 8.715e-10, p corr. =7.0 e-9					

ANOVA table

	SS	*df*	*F*	*p*	*p corr.*
Intercept (NC)	0.004	1	0.246	0.622	
PD cognitive classification	0.006	2	0.196	0.823	
Single-task reaction time	0.810	1	50.578	**0.000**	**1.78^{e-07}**
Sex	0.006	1	0.384	0.538	
Age	0.004	1	0.263	0.610	
MDS-UPDRS-III	0.021	1	1.307	0.258	
Residuals	0.785	49			

7. Linear Regression Model predicting dual-task interference reaction time by cognitive classification controlled for sex, age, and MDS-UPDRS III

	**Dual-task interference reaction time**			
Predictors	*Beta*	*CI (95%)*	*t-stats*	*p*
Intercept (NC)	-26.18	-67.21 – 14.85	-1.28	0.21
MCI	-1.91	-9.69 – 5.86	-0.49	0.62
SCI	2.57	-5.81 – 10.94	0.62	0.54
Sex	0.43	-6.25 – 7.11	0.13	0.90
MDS-UPDRS III	0.29	-0.26 – 0.85	1.06	0.30
Age	-0.02	-0.32 – 0.29	-0.11	0.91
Observations	56			
R^2 / R^2 adjusted	0.041 / -0.055			
F(5,50) = 0.43, p = 0.83				

ANOVA table

	SS	*df*	*F*	*p*
Intercept (NC)	227.282	1	1.643	0.206
PD cognitive classification	143.604	2	0.519	0.598
Sex	2.331	1	0.017	0.897
Age	154.899	1	1.119	0.295
MDS-UPDRS-III	1.688	1	0.012	0.913
Residuals	6918.245	50		

8. Linear Regression Model predicting TUG dual-task by cognitive classification controlled for TUG, sex, age, and MDS-UPDRS III

	**TUG dual-task**				
Predictors	*Beta*	*CI (95%)*	*t-stats*	*p*	*p corr.*
Intercept (NC)	-1.80	-15.15 – 11.55	-0.27	0.79	
SCI	-2.82	-11.80 – 6.17	-0.63	0.53	
MCI	-10.02	-17.39 – -2.65	-2.74	**0.01**	0.18

TUG	1.04	0.56 – 1.52	4.35	**<0.01**	**0.0009**
Sex	2.21	0.06 – 4.36	2.07	**0.04**	0.4
Age	0.16	-0.04 – 0.37	1.59	0.12	
MDS-UPDRS III	-0.27	-0.49 – -0.06	-2.59	**0.01**	0.16
SCI*MDS-UPDRS III	0.20	-0.14 – 0.53	1.18	0.24	
MCI*MDS-UPDRS III	0.38	0.14 – 0.63	3.18	**<0.01**	**0.039**
Observations	55				
R^2 / R^2adjusted	0.583 / 0.511				
F(8,46) = 8.04, p = 1.025e-06, p corr. = 3.4 e-06					

ANOVA table

	SS	***df***	***F***	***p***	***p corr.***
Intercept (NC)	0.960	1	0.074	0.787	
PD cognitive classification	112.003	2	4.310	**0.019**	0.24
TUG	246.392	1	18.963	**0.000**	**0.001**
Sex	55.680	1	4.285	**0.044**	0.44
Age	32.768	1	2.522	0.119	
MDS-UPDRS-III	87.118	1	6.705	**0.013**	0.16
PD cognitive classification*MDS-UPDRS-III	138.384	2	5.325	**0.008**	0.1
Residuals	597.683	46			

9. Linear Regression Model predicting TUG difference by cognitive classification controlled for sex, age, and MDS-UPDRS III

	**TUG Difference**			
Predictors	*Beta*	*CI (95%)*	*t-stats*	*p*
Intercept (NC)	6.37	-7.23 – 19.98	0.94	0.35
SCI	-2.60	-5.39 – 0.18	-1.88	0.07
MCI	-1.10	-3.69 – 1.48	-0.86	0.40
Sex	-1.52	-3.75 – 0.71	-1.37	0.18
MDS-UPDRS III	-0.13	-0.32 – 0.06	-1.41	0.17
Age	-0.01	-0.11 – 0.09	-0.16	0.88
Observations	55			
R^2 / R^2adjusted	0.126 / 0.037			
F(5,49) = 1.42, p = 0.24				

ANOVA table

	SS	***df***	***F***	***p***
Intercept (NC)	13.357	1	0.887	0.351
PD cognitive classification	53.229	2	1.767	0.182
Sex	28.190	1	1.872	0.178
Age	29.768	1	1.976	0.166
MDS-UPDRS-III	0.372	1	0.025	0.876
Residuals	738.014	49		

10. Local maxima from voxel-based morphometry analysis and voxel-wise regression

	Cluster-level p (FWE)	*k*	*T*	*Z*	*Peak-level p (unc)*	*x.y.z {mm}*
PD-MCI<HC	0.150	1086	4.25	3.95	<0.000	26 16 57
			4.22	3.92	<0.000	30 12 46
			4.13	3.85	<0.000	33 14 32
	0.214	857	4.24	3.94	<0.000	-4 48 33
	0.225	823	4.19	3.89	<0.000	-69 -39 3
			3.99	3.73	<0.000	-68 -46 8
			3.66	3.46	<0.000	-68 -30 12
	0.326	595	4.04	3.77	<0.000	-58 -60 33
			3.56	3.37	<0.000	-39 -62 54
			3.46	3.28	0.001	-48 -62 50
	0.728	115	4.02	3.75	<0.000	66 -6 -10
	0.606	223	3.82	3.59	<0.000	-24 64 -12
			3.68	3.47	<0.000	-18 63 -18
PD-MCI>HC	0.460	387	4.09	3.81	<0.000	-9 -104 -10
PD-SCI<HC	0.282	673	4.30	3.93	<0.000	54 -60 -6
			3.45	3.24	0.001	58 -64 9
	0.126	1175	4.25	3.90	<0.000	-68 -45 4
			3.88	3.60	<0.000	-57 -50 -2
			3.81	3.54	<0.000	-54 -42 -9
	0.728	120	3.67	3.43	<0.000	-48 -69 -9
PD- dual-task gait speed	0.014	2992	5.1	4.56	<0.000	21 -39 -28
			4.47	4.08	<0.000	14 -62 -26
			3.84	3.58	<0.000	30 -50 -33

11. Linear Regression Model predicting dual-task gait speed by NBM volume controlled for cognitive classification (interaction term), single-task gait speed, sex, age, and MD-UPDRS III

	**Dual-task gait speed by NBM**				
Predictors	*Beta*	*CI (95%)*	*t-stats*	*p*	*p corr.*
Intercept (NC)	0.83	-0.24 – 1.90	1.57	0.12	

NBM	-0.58	-2.25 – 1.08	-0.70	0.48	
SCI	-1.13	-2.69 – 0.43	-1.46	0.15	
MCI	0.27	-0.77 – 1.32	0.53	0.60	
Age	-0.00	-0.01 – 0.00	-0.90	0.37	
Single-task gait speed	0.77	0.56 – 0.98	7.32	**<0.001**	**1.062e-07**
Sex	-0.01	-0.08 – 0.06	-0.31	0.75	
MDS-UPDRS III	-0.00	-0.01 – 0.00	-1.52	0.13	
SCI*NBM	2.40	-0.75 – 5.56	1.54	0.13	
MCI*NBM	-0.71	-2.90 – 1.48	-0.66	0.52	
Observations	56				

R^2 / R^2adjusted 0.7277/ 0.6744

F(9,46) = 13.66, p = 2.45e-10, p corr. =3.2 e-9

ANOVA table

	SS	*df*	*F*	*p*	*p corr.*
Intercept (NC)	0.039	1	2.465	0.123	
NBM	0.008	1	0.496	0.485	
PD cognitive classification	0.054	2	1.704	0.193	
Age	0.013	1	0.814	0.372	
Single-task gait speed	0.857	1	53.617	**0.000**	**1.062e-07**
Sex	0.002	1	0.099	0.754	
MDS-UPDRS-III	0.037	1	2.315	0.135	
PD cognitive classification*NBM	0.066	2	2.060	0.139	
Residuals	0.735	46			

12. Linear Regression Model predicting dual-task interference gait speed by NBM volume controlled for cognitive classification (interaction term), single-task gait speed, sex, age, and MDS-UPDRS III

	**Dual-task interference gait speed by NBM**			
Predictors	*Beta*	*CI (95%)*	*t-stats*	*p*
Intercept (NC)	35.07	-49.49 – 119.63	0.83	0.41
NBM	-64.14	-206.99 – 78.72	-0.90	0.37
SCI	-71.30	-203.16 – 60.56	-1.09	0.28
MCI	12.84	-76.37 – 102.05	0.29	0.77
Sex	-0.77	-7.06 – 5.52	-0.25	0.81
Age	-0.05	-0.63 – 0.53	-0.18	0.85

MDS-UPDRS III	-0.14	-0.43 – 0.14	-1.01	0.32
SCI*NBM	156.61	-110.61 – 423.82	1.18	0.24
MCI*NBM	-36.30	-222.71 – 150.10	-0.39	0.70
Observations	55			
R^2 / R^2adjusted 0.227 / 0.093				
F(8,46) = 1.69, p = 0.13				

ANOVA table

	SS	*df*	*F*	*p*
Intercept (NC)	82.322	1	0.697	0.408
NBM	96.468	1	0.817	0.371
PD cognitive classification	211.300	2	0.894	0.416
Sex	7.189	1	0.061	0.806
Age	4.001	1	0.034	0.855
MDS-UPDRS-III	119.342	1	1.010	0.320
PD cognitive classification*NBM	271.657	2	1.150	0.326
Residuals	5433.762	46		

13. Linear Regression Model predicting dual-task gait speed by DLPFC volume controlled for cognitive classification (interaction term), single-task gait speed, sex, age, and MDS-UPDRS III

Dual-task gait speed by DLPFC

Predictors	*Beta*	*CI (95%)*	*t-stats*	*p*	*p corr.*
Intercept (NC)	-0.53	-1.72 – 0.66	-0.90	0.37	
DLPFC	2.17	-0.39 – 4.72	1.71	0.09	
SCI	0.27	-1.23 – 1.77	0.36	0.72	
MCI	0.88	-0.18 – 1.95	1.67	0.10	
Age	0.00	-0.01 – 0.01	0.38	0.71	
Single-task gait speed	0.80	0.57 – 1.04	6.96	**<0.001**	**1.062e-07**
Sex	-0.01	-0.08 – 0.07	-0.25	0.80	
MDS-UPDRS III	-0.00	-0.01 – 0.00	-1.44	0.16	
SCI*DLPFC	-0.59	-5.14 – 3.97	-0.26	0.80	
MCI*DLPFC	-2.86	-6.09 – 0.36	-1.79	0.08	
Observations	56				
R^2 / R^2adjusted	0.719 / 0.664				
F(9,46) = 13.08, p = 4.828e-10, p corr. =4.8e-9					

ANOVA table

	SS	*df*	*F*	*p*	*p corr.*
Intercept (NC)	0.013	1	0.811	0.373	
DLPFC	0.048	1	2.915	0.094	
PD cognitive classification	0.048	2	1.452	0.245	
Age	0.002	1	0.144	0.706	
Single-task gait speed	0.798	1	48.381	**0.000**	**1.062e-07**
Sex	0.001	1	0.064	0.802	
MDS-UPDRS-III	0.034	1	2.086	0.155	
PD cognitive classification*DLPFC	0.057	2	1.722	0.190	
Residuals	0.759	46			

14. Linear Regression Model predicting dual-task interference gait speed by DLPFC volume controlled for cognitive classification (interaction term, sex, age, and MDS-UPDRS III

	**Dual-task interference gait speed by DLPFC**			
Predictors	*Beta*	*CI (95%)*	*t-stats*	*p*
Intercept (NC)	-71.61	-166.37 – 23.15	-1.52	0.14
DLPFC	164.47	-53.27 – 382.20	1.52	0.14
SCI	47.18	-76.57 – 170.93	0.77	0.45
MCI	78.41	-11.38 – 168.19	1.76	0.09
Sex	-0.36	-6.76 – 6.03	-0.11	0.91
Age	0.26	-0.31 – 0.83	0.92	0.36
MDS-UPDRS III	-0.17	-0.48 – 0.14	-1.11	0.27
SCI*DLPFC	-123.82	-500.28 – 252.63	-0.66	0.51
MCI*DLPFC	-250.61	-522.46 – 21.25	-1.86	0.07
Observations	55			
R^2 / R^2adjusted 0.218 / 0.082				
F(8.46) = 1.60. p = 0.15				

ANOVA table

	SS	*df*	*F*	*p*
Intercept (NC)	276.846	1	2.314	0.135
DLPFC	276.559	1	2.312	0.135
PD cognitive classification	371.050	2	1.551	0.223
Sex	1.569	1	0.013	0.909
Age	100.284	1	0.838	0.365
MDS-UPDRS-III	146.288	1	1.223	0.275
PD cognitive classification*DLPFC	412.127	2	1.722	0.190
Residuals	5503.067	46		

15. Linear Regression Model predicting dual-task gait speed by hippocampus volume controlled for cognitive classification (interaction term), single-task gait speed, sex, age, and MDS-UPDRS III

	**Dual-task gait speed by hippocampus**				
Predictors	*Beta*	*CI (95%)*	*t-stats*	*p*	*p corr.*
Intercept (NC)	0.41	-0.63 – 1.44	0.79	0.43	
hippocampus	-0.09	-1.17 – 1.00	-0.16	0.87	
SCI	-0.37	-1.45 – 0.71	-0.69	0.49	
MCI	-0.09	-0.98 – 0.80	-0.20	0.85	
Age	-0.00	-0.01 – 0.01	-0.24	0.81	
Single-task gait speed	0.78	0.56 – 1.01	6.99	**<0.001**	**1.062e-07**
Sex	-0.01	-0.09 – 0.07	-0.27	0.79	
MDS-UPDRS III	-0.00	-0.01 – 0.00	-1.26	0.21	
SCI*hippocampus	0.71	-1.06 – 2.49	0.81	0.42	
MCI*hippocampus	0.04	-1.42 – 1.51	0.06	0.96	
Observations	56				
R^2 / R^2 adjusted	0.700 / 0.641				
F(9,46) = 11.9, p = 1.982e-09, p corr. = 1.3e-08					

ANOVA table

	SS	*df*	*F*	*p*	*p corr.*
Intercept (NC)	0.011	1	0.628	0.432	
Hippocampus	0.000	1	0.025	0.875	
PD cognitive classification	0.008	2	0.241	0.787	
Age	0.001	1	0.060	0.808	
Single-task gait	0.860	1	48.841	**0.000**	**1.062e-07**
Sex	0.001	1	0.074	0.787	
MDS-UPDRS-III	0.028	1	1.586	0.214	
PD cognitive classification*Hippocampus	0.013	2	0.365	0.696	
Residuals	0.810	46			

16. Linear Regression Model predicting dual-task interference gait speed by hippocampus volume controlled for cognitive classification (interaction term), sex, age, and MDS-UPDRS III

	**Dual-task interference gait speed by hippocampus**			
Predictors	*Beta*	*CI (95%)*	*t-stats*	*p*
Intercept (NC)	10.57	-70.10 – 91.23	0.26	0.79

Hippocampus	-26.99	-123.31 – 69.33	-0.56	0.58
SCI	-16.09	-106.35 – 74.18	-0.36	0.72
MCI	-20.30	-97.23 – 56.64	-0.53	0.60
Sex	-0.55	-7.14 – 6.03	-0.17	0.87
Age	0.08	-0.52 – 0.68	0.26	0.79
MDS-UPDRS III	-0.13	-0.43 – 0.18	-0.85	0.40
SCI*Hippocampus	36.61	-112.86 – 186.08	0.49	0.62
MCI*Hippocampus	27.16	-99.78 – 154.09	0.43	0.67
Observations	55			
R^2 / R^2 adjusted 0.165 / 0.020				
F(8,46) = 1.14, p = 0.36				

ANOVA table

	SS	***df***	***F***	***p***
Intercept (NC)	8.873	1	0.070	0.793
Hippocampus	40.616	1	0.318	0.575
PD cognitive classification	38.649	2	0.151	0.860
Sex	3.656	1	0.029	0.866
Age	8.952	1	0.070	0.792
MDS-UPDRS-III	91.664	1	0.718	0.401
PD cognitive classification*Hippocampus	38.194	2	0.150	0.861
Residuals	5870.873	46		

17. Linear Regression Model predicting dual-task gait speed by cerebellum volume controlled for cognitive classification (interaction term), single-task gait speed, sex, age, and MDS-UPDRS III

	**Dual-task gait speed by cerebellum**				
Predictors	*Beta*	*CI (95%)*	*t-stats*	*p*	*p corr.*
Intercept (NC)	-0.76	-1.96 – 0.43	-1.29	0.20	
Cerebellum	2.05	-0.06 – 4.15	1.96	0.06	
SCI	0.07	-1.22 – 1.36	0.11	0.91	
MCI	0.01	-1.13 – 1.15	0.02	0.99	
Age	0.00	-0.00 – 0.01	0.71	0.48	
Single-task gait speed	0.74	0.53 – 0.95	7.04	**<0.001**	**1.06e-07**
Sex	-0.02	-0.09 – 0.05	-0.67	0.50	

MDS-UPDRS III	-0.00	-0.01 – 0.00	-0.96	0.34
SCI*Cerebellum	-0.12	-2.84 – 2.60	-0.09	0.93
MCI*Cerebellum	-0.14	-2.65 – 2.37	-0.11	0.91
Observations	56			
R^2 / R^2 adjusted	0.766 / 0.720			
F(9,46) = 16.70, p = 9.175e-12, p corr. =3.7e-10				

ANOVA table

	SS	*df*	*F*	*p*	*p corr.*
Intercept (NC)	0.023	1	1.661	0.204	
Cerebellum	0.053	1	3.841	0.056	
PD cognitive classification	0.000	2	0.007	0.993	
Age	0.007	1	0.508	0.479	
Single-task gait	0.682	1	49.606	**0.000**	**1.06e-07**
Sex	0.006	1	0.454	0.504	
MDS-UPDRS-III	0.013	1	0.913	0.344	
PD cognitive classification*Cerebellum	0.000	2	0.007	0.993	
Residuals	0.633	46			

18. Linear Regression Model predicting dual-task interference gait speed by cerebellum volume controlled for cognitive classification (interaction term), single-task gait speed, sex, age, and MDS-UPDRS III

Dual-task interference gait speed by cerebellum

Predictors	*Beta*	*CI (95%)*	*t-stats*	*p*	*p corr.*
Intercept (NC)	-116.99	-226.13 – -7.85	-2.16	**0.04**	**0.362**
Cerebellum	181.76	-8.59 – 372.10	1.92	0.06	
SCI	54.80	-59.54 – 169.14	0.96	0.34	
MCI	3.39	-96.12 – 102.90	0.07	0.95	
Sex	-1.70	-7.70 – 4.29	-0.57	0.57	
Age	0.44	-0.10 – 0.98	1.65	0.11	
MDS-UPDRS III	-0.03	-0.31 – 0.24	-0.23	0.82	
SCI*Cerebellum	-108.48	-351.29 – 134.33	-0.90	0.37	
MCI*Cerebellum	-13.94	-232.37 – 204.50	-0.13	0.90	
Observations	55				
R^2 / R^2 adjusted	0.317 / 0.198				
F(8,46) = 2.67, p = 0.01711					

ANOVA table

	SS	***df***	***F***	***p***	***p corr.***
Intercept (NC)	486.416	1	4.656	**0.036**	0.36
Cerebellum	385.966	1	3.694	0.061	
PD cognitive classification	143.406	2	0.686	0.509	
Sex	34.129	1	0.327	0.570	
Age	285.104	1	2.729	0.105	
MDS-UPDRS-III	5.690	1	0.054	0.817	
PD cognitive classification*Cerebellum	117.204	2	0.561	0.575	
Residuals	4805.951	46			

19. Linear Regression Model predicting dual-task step-time variability by NBM volumes controlled for cognitive classification (interaction term), total intracranial volume, single-task step-time variability, sex, age, and MDS-UPDRS III

	**Dual-task step-time variability by NBM**				
Predictors	*Beta*	*CI (95%)*	*t-stats*	*p*	*p corr.*
Intercept (NC)	0.01	-0.04 – 0.06	0.35	0.73	
NBM	-0.01	-0.10 – 0.08	-0.19	0.85	
SCI	0.01	-0.08 – 0.09	0.18	0.86	
MCI	-0.03	-0.09 – 0.03	-1.04	0.30	
Age	-0.00	-0.00 – 0.00	-0.27	0.79	
Single-task step-time variability	0.76	0.31 – 1.21	3.42	**<0.001**	**0.014**
Sex	0.00	-0.00 – 0.01	0.62	0.54	
MDS-UPDRS III	0.00	-0.00 – 0.00	1.33	0.19	
SCI*NBM	-0.02	-0.19 – 0.15	-0.19	0.85	
MCI*NBM	0.07	-0.05 – 0.20	1.25	0.22	
Observations	55				

R^2 / R^2adjusted 0.505 / 0.406

F(9,45) = 5.10, p = 9.312e-05, p corr. =0.0002

ANOVA table

	SS	*df*	*F*	*p*	*p corr.*
Intercept (NC)	0.000	1	0.122	0.729	
NBM	0.000	1	0.035	0.852	
PD cognitive classification	0.000	2	0.731	0.487	
Age	0.000	1	0.070	0.792	
Single-task step-time variability	0.001	1	11.667	**0.001**	**0.014**
Sex	0.000	1	0.381	0.540	
MDS-UPDRS-III	0.000	1	1.780	0.189	
PD cognitive classification*NBM	0.000	2	1.035	0.363	
Residuals	0.002	45			

20. Linear Regression Model predicting dual-task interference step-time variability by NBM volumes controlled for cognitive classification (interaction term), total intracranial volume, sex, age, and MDS-UPDRS III

	**Dual-task interference step-time variability by NBM**			
Predictors	*Beta*	*CI (95%)*	*t-stats*	*p*
Intercept (NC)	73.95	-261.30 – 409.20	0.44	0.66
NBM	-11.66	-578.01 – 554.70	-0.04	0.97
SCI	56.16	-466.60 – 578.91	0.22	0.83
MCI	-137.00	-490.68 – 216.68	-0.78	0.44
Age	12.69	-12.24 – 37.63	1.02	0.31
Sex	-1.05	-3.34 – 1.24	-0.92	0.36
MDS-UPDRS III	0.61	-0.53 – 1.75	1.07	0.29
SCI*NBM	-122.63	-1182.03 – 936.76	-0.23	0.82
MCI*NBM	351.75	-387.26 – 1090.75	0.96	0.34
Observations	55			
R^2 / R^2adjusted	0.234 / 0.101			
F(8.46) = 1.76. p = 0.11				

ANOVA table

	SS	*df*	*F*	*p*
Intercept (NC)	366.016	1	0.197	0.659
NBM	3.186	1	0.002	0.967
PD cognitive classification	1723.804	2	0.464	0.632
Sex	1950.061	1	1.050	0.311
Age	1586.526	1	0.855	0.360
MDS-UPDRS-III	2122.856	1	1.143	0.291
PD cognitive classification*NBM	2564.056	2	0.691	0.506
Residuals	85406.298	46		

21. Linear Regression Model predicting dual-task step-time variability by DLPFC volumes controlled for cognitive classification (interaction term), total intracranial volume, single-task step-time variability, sex, age (interaction term), and MDS-UPDRS III

	**Dual-task step-time variability by DLPFC**					
Predictors	*Beta*	*CI (95%)*	*t-stats*	*std. t-stats*	*p*	*p corr.*
Intercept (NC)	0.51	0.22 – 0.81	3.51	-0.24	**<0.001**	**0.042**
DLPFC	-1.56	-2.49 – -0.64	-3.39	-1.55	**<0.001**	0.058
SCI	-0.03	-0.10 – 0.05	-0.69	-0.61	0.50	
MCI	0.01	-0.04 – 0.06	0.33	1.82	0.74	
Age	-0.01	-0.01 – -0.00	-3.32	0.19	**<0.001**	0.072
Single-task step-time variability	0.61	0.18 – 1.04	2.84	2.84	**0.01**	**0.05**
Sex	0.00	-0.00 – 0.00	0.31	0.31	0.76	
MDS-UPDRS III	0.00	-0.00 – 0.00	1.16	1.16	0.25	
SCI*DLPFC	0.07	-0.15 – 0.30	0.65	0.65	0.52	
MCI*DLPFC	-0.01	-0.17 – 0.15	-0.18	-0.18	0.86	
DLPFC*Age	0.02	0.01 – 0.03	3.22	3.22	**<0.001**	0.10
Observations	55					
R^2 / R^2adjusted 0.607 / 0.517						
F(10,44) = 6.78, p = 2.773e-06, p corr. =8.0e-06						

ANOVA table

	SS	*df*	*F*	*p*	*p corr.*
Intercept (NC)	0.000	1	12.306	**0.001**	**0.042**
DLPFC	0.000	1	11.522	**0.001**	0.058
PD cognitive classification	0.000	2	0.435	0.650	
Age	0.000	1	11.014	**0.002**	0.072
Single-task step-time variability	0.000	1	8.049	**0.007**	**0.05**
Sex	0.000	1	0.099	0.755	
MDS-UPDRS-III	0.000	1	1.355	0.251	
PD cognitive classification*DLPFC	0.000	2	0.313	0.733	
Age*DLPFC	0.000	1	10.359	**0.002**	**0.10**
Residuals	0.002	44			

22. Linear Regression Model predicting step time variability dual-task by DLPFC volumes controlled for cognitive classification (interaction term), total intracranial volume, step time variability single-task, sex, age, and MDS-UPDRS III

	**Step time variability dual-task interference by DLPFC**			
Predictors	*Beta*	*CI (95%)*	*t-stats*	*p*
Intercept (NC)	342.91	-30.02 – 715.83	1.85	0.07
DLPFC	-585.33	-1442.24 – 271.59	-1.37	0.18
SCI	-152.40	-639.43 – 334.62	-0.63	0.53
MCI	-119.63	-472.98 – 233.73	-0.68	0.50
Sex	11.76	-13.40 – 36.93	0.94	0.35
Age	-2.15	-4.39 – 0.09	-1.93	0.06
MDS-UPDRS III	0.50	-0.71 – 1.72	0.84	0.41
SCI*DLPFC	435.66	-1045.91 – 1917.23	0.59	0.56
MCI*DLPFC	452.65	-617.24 – 1522.54	0.85	0.40
Observations	55			
R^2 / R^2adjusted 0.236 / 0.103				
F(8,46) = 1.77, p = 0.11				

ANOVA table

	**SS**	**df**	**F**	**p**
Intercept (NC)	6347.742	1	3.426	0.071
DLPFC	3502.899	1	1.890	0.176
PD cognitive classification	1158.808	2	0.313	0.733
Sex	1640.051	1	0.885	0.352
Age	6907.860	1	3.728	0.060
MDS-UPDRS-III	1304.379	1	0.704	0.406
PD cognitive classification*DLPFC	1501.460	2	0.405	0.669
Residuals	85235.192	46		

23. Linear Regression Model predicting dual-task step-time variability by hippocampus volumes controlled for cognitive classification (interaction term), total intracranial volume, single-task step-time variability, sex, age, and MDS-UPDRS III

	**Dual-task step-time variability by hippocampus**				
Predictors	*Beta*	*CI (95%)*	*t-stats*	*p*	*p corr.*
Intercept (NC)	0.02	-0.03 – 0.07	0.64	0.53	

Hippocampus	-0.00	-0.06 – 0.06	-0.08	0.94	
SCI	0.01	-0.04 – 0.07	0.48	0.64	
MCI	0.00	-0.05 – 0.05	0.14	0.89	
Single-task step-time variability	0.78	0.33 – 1.24	3.46	**<0.001**	**0.014**
Sex	0.00	-0.00 – 0.01	0.65	0.52	
Age	-0.00	-0.00 – 0.00	-0.92	0.36	
MDS-UPDRS III	0.00	-0.00 – 0.00	1.20	0.23	
SCI*Hippocampus	-0.02	-0.12 – 0.07	-0.51	0.62	
MCI*Hippocampus	0.00	-0.08 – 0.08	0.09	0.93	
Observations	55				
R^2 / R^2 adjusted	0.478 / 0.374				

F(9,45) = 4.59, p = 0.0002524, p corr. =0.0005

ANOVA table

	SS	*df*	*F*	*p*	*p corr.*
Intercept (NC)	0.000	1	0.410	0.525	
Hippocampus	0.000	1	0.006	0.940	
PD cognitive classification	0.000	2	0.116	0.891	
Single-task step-time variability	0.001	1	12.002	**0.001**	**0.014**
Sex	0.000	1	0.421	0.520	
Age	0.000	1	0.838	0.365	
MDS-UPDRS-III	0.000	1	1.451	0.235	
PD cognitive classification*Hippocampus	0.000	2	0.184	0.833	
Residuals	0.002	45			

24. Linear Regression Model predicting dual-task step-time variability by hippocampus volumes controlled for cognitive classification (interaction term), total intracranial volume, sex, age, and MDS-UPDRS III

	**Dual-task interference step-time variability by hippocampus**			
Predictors	*Beta*	*CI (95%)*	*t-stats*	*p*
Intercept (NC)	83.80	-228.17 – 395.77	0.54	0.59
Hippocampus	45.42	-327.10 – 417.95	0.25	0.81
SCI	125.33	-223.76 – 474.43	0.72	0.47
MCI	44.06	-253.48 – 341.61	0.30	0.77
Sex	13.40	-12.08 – 38.88	1.06	0.30
Age	-1.65	-3.96 – 0.66	-1.44	0.16

MDS-UPDRS III	0.60	-0.57 – 1.77	1.04	0.30
SCI*Hippocampus	-219.22	-797.33 – 358.88	-0.76	0.45
MCI*Hippocampus	-24.54	-515.49 – 466.40	-0.10	0.92

Observations 55

R^2 / R^2 adjusted 0.213 / 0.076

F(8,46) = 1.55, p = 0.17

ANOVA table

	SS	***df***	***F***	***p***
Intercept (NC)	558.139	1	0.292	0.591
Hippocampus	115.003	1	0.060	0.807
PD cognitive classification	997.433	2	0.261	0.771
Sex	2140.228	1	1.121	0.295
Age	3943.947	1	2.066	0.157
MDS-UPDRS-III	2059.424	1	1.079	0.304
PD cognitive classification*Hippocampus	1245.471	2	0.326	0.723
Residuals	87818.363	46		

25. Linear Regression Model predicting dual-task step-time variability by cerebellum volumes controlled for cognitive classification (interaction term), total intracranial volume, single-task step-time variability, sex, age, and MDS-UPDRS III

	**Dual-task step-time variability by cerebellum**				
Predictors	*Beta*	*CI (95%)*	*t-stats*	*p*	*p corr.*
Intercept (NC)	0.10	0.03 – 0.17	2.82	**0.01**	0.10
Cerebellum	-0.15	-0.27 – -0.02	-2.41	**0.02**	0.4
SCI	-0.04	-0.12 – 0.03	-1.22	0.23	
MCI	-0.03	-0.09 – 0.04	-0.81	0.42	
Single-task step-time variability	0.77	0.34 – 1.20	3.59	**<0.001**	**0.014**
Sex	0.00	-0.00 – 0.01	0.95	0.35	
Age	-0.00	-0.00 – 0.00	-2.01	**0.05**	0.6
MDS-UPDRS III	0.00	-0.00 – 0.00	0.67	0.51	
SCI*Cerebellum	0.10	-0.06 – 0.25	1.25	0.22	
MCI*Cerebellum	0.07	-0.07 – 0.21	0.96	0.34	

Observations 55

R^2 / R^2 adjusted 0.566 / 0.479

F(9,45) = 6.52, p = 6.965e-06, p corr. =1.9e-05

ANOVA table

	SS	*df*	*F*	*p*	*p corr.*
Intercept (NC)	0.000	1	7.931	**0.007**	0.1
Cerebellum	0.000	1	5.805	**0.020**	0.4
PD cognitive classification	0.000	2	0.750	0.478	
Single-task step-time variability	0.001	1	12.917	**0.001**	**0.014**
Sex	0.000	1	0.898	0.348	
Age	0.000	1	4.032	0.051	0.6
MDS-UPDRS-III	0.000	1	0.443	0.509	
PD cognitive classification*Cerebellum	0.000	2	0.820	0.447	
Residuals	0.002	45			

26. Linear Regression Model predicting dual-task interference step-time variability by cerebellum volumes controlled for cognitive classification (interaction term), total intracranial volume, sex, age, and MDS-UPDRS II

	Dual-task interference step time variability by cerebellum				
Predictors	*Beta*	*CI (95%)*	*t-stats*	*p*	*p corr.*
Intercept (NC)	636.06	215.98 – 1056.14	3.05	**<0.001**	0.08
Cerebellum	-909.17	-1641.81 – -176.53	-2.50	**0.02**	0.4
SCI	-297.99	-738.08 – 142.09	-1.36	0.18	
MCI	-123.58	-506.58 – 259.42	-0.65	0.52	
Sex	16.56	-6.52 – 39.63	1.44	0.16	
Age	-3.03	-5.11 – -0.94	-2.93	**0.01**	0.1
MDS-UPDRS III	0.17	-0.89 – 1.23	0.33	0.75	
SCI*cerebellum	640.97	-293.60 – 1575.54	1.38	0.17	
MCI*cerebellum	327.62	-513.14 – 1168.38	0.78	0.44	
Observations	55				
R^2 / R^2 adjusted	0.362 / 0.251				

F(8,46) = 3.26, p = 0.005069, p corr. =0.009

ANOVA table

	SS	*df*	*F*	*p*	*p corr.*
Intercept (NC)	14377.557	1	9.289	**0.004**	0.08
Cerebellum	9657.371	1	6.240	**0.016**	0.4
PD cognitive classification	2963.527	2	0.957	0.391	
Sex	3228.299	1	2.086	0.155	
Age	13254.756	1	8.564	**0.005**	0.1
MDS-UPDRS-III	164.492	1	0.106	0.746	
PD cognitive classification*Cerebellum	2966.869	2	0.958	0.391	
Residuals	71196.934	46			

27. Linear Regression Model predicting dual-task reaction time by NBM volumes controlled for cognitive classification (interaction term), single-task reaction time, sex, age, and MDS-UPDRS-III

	**Dual-task reaction time by NBM**				
Predictors	*Beta*	*CI (95%)*	*t-stats*	*p*	*p corr.*
Intercept (NC)	0.24	-0.87 – 1.35	0.44	0.67	
NBM	-0.41	-2.13 – 1.32	-0.47	0.64	
SCI	0.30	-1.26 – 1.86	0.39	0.70	
MCI	-0.68	-1.73 – 0.38	-1.29	0.20	
Age	0.00	-0.00 – 0.01	0.76	0.45	
Single-task reaction time	0.65	0.46 – 0.84	6.77	**<0.001**	**2.7e-07**
Sex	-0.02	-0.10 – 0.05	-0.60	0.55	
MDS-UPDRS III	0.00	-0.00 – 0.01	1.35	0.18	
SCI*NBM	-0.53	-3.69 – 2.63	-0.34	0.74	
MCI*NBM	1.43	-0.79 – 3.64	1.30	0.20	
Observations	56				
R^2 / R^2adjusted	0.672 / 0.608				
F(9,46) = 10.48, p = 1.337e-08, p corr. =5.9e-08					

ANOVA table

	SS	***df***	***F***	***p***	***p corr.***
Intercept (NC)	0.003	1	0.190	0.665	
NBM	0.004	1	0.224	0.638	
PD cognitive classification	0.043	2	1.337	0.273	
Age	0.009	1	0.584	0.449	
Single-task reaction time	0.736	1	45.774	**0.000**	**2.7e-07**
Sex	0.006	1	0.358	0.552	
MDS-UPDRS-III	0.029	1	1.826	0.183	
PD cognitive classification*NBM	0.043	2	1.324	0.276	
Residuals	0.740	46			

28. Linear Regression Model predicting dual-task interference reaction time by NBM volumes controlled for cognitive classification (interaction term), sex, age, and MDS-UPDRS-III

	**Dual-task interference reaction time by NBM**			
Predictors	*Beta*	*CI (95%)*	*t-stats*	*p*

Intercept (NC)	-47.33	-138.89 – 44.22	-1.04	0.30
NBM	29.26	-125.49 – 184.01	0.38	0.71
SCI	84.42	-58.42 – 227.26	1.19	0.24
MCI	-25.68	-122.32 – 70.96	-0.53	0.60
Sex	0.69	-6.03 – 7.41	0.21	0.84
Age	0.38	-0.24 – 1.00	1.22	0.23
MDS-UPDRS III	0.02	-0.29 – 0.33	0.11	0.91
SCI*NBM	-164.86	-454.30 – 124.58	-1.15	0.26
MCI*NBM	50.11	-151.78 – 252.00	0.50	0.62
Observations	56			
R^2 / R^2adjusted	0.096 / -0.058			
F(8,47) = 0.62, p = 0.76				

ANOVA table

	SS	***df***	***F***	***p***
Intercept (NC)	150.154	1	1.082	0.304
NBM	20.088	1	0.145	0.705
PD cognitive classification	359.803	2	1.296	0.283
Sex	5.973	1	0.043	0.837
Age	207.711	1	1.496	0.227
MDS-UPDRS-III	1.657	1	0.012	0.913
PD cognitive classification*NBM	336.676	2	1.213	0.306
Residuals	6523.585	47		

29. Linear Regression Model predicting dual-task reaction time by DLPFC volumes controlled for cognitive classification (interaction term), single-task reaction time, sex, age, and MDS-UPDRS-III

	**Dual-task reaction time by DLPFC**				
Predictors	*Beta*	*CI (95%)*	*t-stats*	*p*	*p corr.*
Intercept (NC)	0.95	-0.35 – 2.25	1.47	0.15	
DLPFC	-1.72	-4.41 – 0.98	-1.28	0.21	
SCI	0.34	-1.09 – 1.76	0.48	0.64	
MCI	-0.44	-1.59 – 0.70	-0.78	0.44	
Age	-0.00	-0.01 – 0.01	-0.40	0.69	
Single-task reaction time	0.59	0.38 – 0.80	5.54	**<0.001**	**0.00001**

Sex	-0.02	-0.10 – 0.05	-0.62	0.54
MDS-UPDRS III	0.00	-0.00 – 0.01	1.48	0.15
SCI*DLPFC	-0.98	-5.31 – 3.34	-0.46	0.65
MCI*DLPFC	1.37	-2.13 – 4.87	0.79	0.44
Observations	56			
R^2 / R^2adjusted	0.677 / 0.614			

F(9,46) = 10.71, p= 9.837e-09, p corr. =4.9e-08

ANOVA table

	SS	*df*	*F*	*p*	*p corr.*
Intercept (NC)	0.034	1	2.148	0.150	
DLPFC	0.026	1	1.640	0.207	
PD cognitive classification	0.019	2	0.599	0.554	
Age	0.002	1	0.157	0.694	
Single-task reaction time	0.486	1	30.684	**0.000**	**0.00001**
Sex	0.006	1	0.387	0.537	
MDS-UPDRS-III	0.035	1	2.189	0.146	
PD cognitive classification*DLPFC	0.018	2	0.583	0.562	
Residuals	0.729	46			

30. Linear Regression Model predicting dual-task interference reaction time by DLPFC volumes controlled for cognitive classification (interaction term), sex, age, and MDS-UPDRS-III

	**Dual-task interference reaction time by DLPFC**			
Predictors	*Beta*	*CI (95%)*	*t-stats*	*p*
Intercept (NC)	-12.88	-116.12 – 90.37	-0.25	0.80
DLPFC	-26.37	-262.92 – 210.18	-0.22	0.82
SCI	18.01	-116.62 – 152.64	0.27	0.79
MCI	22.31	-75.45 – 120.06	0.46	0.65
Sex	0.59	-6.28 – 7.46	0.17	0.86
Age	0.24	-0.38 – 0.86	0.77	0.44
MDS-UPDRS III	-0.04	-0.38 – 0.29	-0.25	0.80
SCI*DLPFC	-48.15	-457.43 – 361.13	-0.24	0.81
MCI*DLPFC	-74.26	-370.05 – 221.53	-0.51	0.62
Observations	56			
R^2 / R^2adjusted 0.072 / -0.086				

F(8,47) = 0.46, p = 0.88

ANOVA table

	SS	*df*	*F*	*p*
Intercept (NC)	8.963	1	0.063	0.803
DLPFC	7.163	1	0.050	0.824
PD cognitive classification	31.544	2	0.111	0.895
Sex	4.247	1	0.030	0.864
Age	85.014	1	0.597	0.444
MDS-UPDRS-III	9.154	1	0.064	0.801
PD cognitive classification*DLPFC	36.696	2	0.129	0.879
Residuals	6693.354	47		

31. Linear Regression Model predicting dual-task reaction time by hippocampus volumes controlled for cognitive classification (interaction term), single-task reaction time, sex, age, and MDS-UPDRS-III

	**Dual-task reaction time by hippocampus**				
Predictors	*Beta*	*CI (95%)*	*t-stats*	*p*	*p corr.*
Intercept (NC)	0.62	-0.45 – 1.69	1.17	0.25	
hippocampus	-0.65	-1.75 – 0.45	-1.19	0.24	
SCI	-0.10	-1.10 – 0.90	-0.19	0.85	
MCI	-0.60	-1.45 – 0.26	-1.40	0.17	
Age	0.00	-0.01 – 0.01	0.14	0.89	
Single-task reaction time	0.62	0.41 – 0.82	6.04	**<0.001**	**2.5e-6**
Sex	-0.02	-0.10 – 0.05	-0.62	0.54	
MDS-UPDRS III	0.00	-0.00 – 0.01	1.43	0.16	
SCI*hippocampus	0.21	-1.45 – 1.86	0.25	0.80	
MCI*hippocampus	1.00	-0.41 – 2.42	1.43	0.16	
Observations	56				
R^2 / R^2 adjusted	0.669 / 0.605				

F(9,46) = 10.35, p = 1.603e-08, p corr. =6.4e-08

ANOVA table

	SS	*df*	*F*	*p*	*p corr.*
Intercept (NC)	0.022	1	1.358	0.250	
Hippocampus	0.023	1	1.415	0.240	
PD cognitive classification	0.035	2	1.066	0.353	
Age	0.000	1	0.019	0.890	
Single-task reaction time	0.591	1	36.464	**0.000**	**2.5e-6**
Sex	0.006	1	0.380	0.540	
MDS-UPDRS-III	0.033	1	2.034	0.161	
PD cognitive classification*Hippocampus	0.035	2	1.085	0.346	
Residuals	0.746	46			

32. Linear Regression Model predicting dual-task interference reaction time by hippocampus volumes controlled for cognitive classification (interaction term), sex, age, and MDS-UPDRS-III

	**Dual-task interference reaction time by hippocampus**			
Predictors	*Beta*	*CI (95%)*	*t-stats*	*p*
Intercept (NC)	-25.81	-109.82 – 58.20	-0.62	0.54
Hippocampus	-11.41	-109.41 – 86.60	-0.23	0.82
SCI	-8.50	-102.69 – 85.69	-0.18	0.86
MCI	-37.57	-117.77 – 42.64	-0.94	0.35
Sex	0.63	-6.25 – 7.51	0.18	0.86
Age	0.37	-0.26 – 1.01	1.18	0.24
MDS-UPDRS III	0.01	-0.31 – 0.33	0.09	0.93
SCI*Hippocampus	18.82	-136.70 – 174.35	0.24	0.81
MCI*Hippocampus	58.60	-73.32 – 190.52	0.89	0.38
Observations	56			
R^2 / R^2 adjusted	0.062 / -0.098			
F(8,47) = 0.39, p = 0.92				

ANOVA table

	SS	*df*	*F*	*p*
Intercept (NC)	54.983	1	0.382	0.540
Hippocampus	7.892	1	0.055	0.816
PD cognitive classification	135.054	2	0.469	0.628
Sex	4.825	1	0.034	0.856
Age	200.240	1	1.391	0.244
MDS-UPDRS-III	1.124	1	0.008	0.930
PD cognitive classification*Hippocampus	117.923	2	0.410	0.666
Residuals	6764.820	47		

33. Linear Regression Model predicting dual-task reaction time by cerebellum volumes controlled for cognitive classification (interaction term), single-task reaction time, sex, age, and MDS-UPDRS-III

	**Dual-task reaction time by cerebellum**				
Predictors	*Beta*	*CI (95%)*	*t-stats*	*p*	*p corr.*
Intercept (NC)	0.57	-0.77 – 1.91	0.86	0.39	
cerebellum	-0.63	-2.86 – 1.59	-0.57	0.57	
SCI	0.33	-1.03 – 1.68	0.49	0.63	
MCI	0.28	-0.92 – 1.48	0.47	0.64	

Age	-0.00	-0.01 – 0.01	-0.16	0.87	
Single-task reaction time	0.66	0.46 – 0.85	6.92	**<0.001**	**2.38e-7**
Sex	-0.01	-0.09 – 0.06	-0.31	0.76	
MDS-UPDRS III	0.00	-0.00 – 0.01	0.90	0.37	
SCI*cerebellum	-0.60	-3.48 – 2.28	-0.42	0.68	
MCI*cerebellum	-0.63	-3.28 – 2.02	-0.48	0.64	
Observations	56				
R^2 / R^2 adjusted	0.683 / 0.621				

$F(9,46) = 11.02$, $p = 6.505e-09$, p corr. =3.7e-08

ANOVA table

	SS	*df*	*F*	*p*	*p corr.*
Intercept (NC)	0.012	1	0.743	0.393	
Cerebellum	0.005	1	0.328	0.570	
PD cognitive classification	0.005	2	0.146	0.865	
Age	0.000	1	0.025	0.874	
Single-task reaction time	0.745	1	47.920	**0.000**	**2.38e-7**
Sex	0.002	1	0.098	0.756	
MDS-UPDRS-III	0.013	1	0.805	0.374	
PD cognitive classification*Cerebellum	0.004	2	0.128	0.881	
Residuals	0.715	46			

34. Linear Regression Model predicting dual-task interference reaction time by cerebellum volumes controlled for cognitive classification (interaction term), sex, age, and MDS-UPDRS-III

	**Dual-task interference reaction time by cerebellum**			
Predictors	*Beta*	*CI (95%)*	*t-stats*	*p*
Intercept (NC)	-8.03	-124.60 – 108.54	-0.14	0.89
cerebellum	-16.31	-220.59 – 187.96	-0.16	0.87
SCI	41.11	-85.48 – 167.70	0.65	0.52
MCI	42.77	-66.63 – 152.17	0.79	0.44
Sex	1.51	-5.25 – 8.27	0.45	0.65
Age	0.15	-0.46 – 0.76	0.50	0.62
MDS-UPDRS III	-0.05	-0.36 – 0.26	-0.32	0.75
SCI*cerebellum	-79.64	-348.71 – 189.43	-0.60	0.55
MCI*cerebellum	-99.56	-340.35 – 141.22	-0.83	0.41

Observations	56
R^2 / R^2 adjusted	0.111 / -0.041
F(8,47) = 0.73, p = 0.66	

ANOVA table

	SS	*df*	*F*	*p*
Intercept (NC)	2.618	1	0.019	0.890
Cerebellum	3.523	1	0.026	0.873
PD cognitive classification	92.029	2	0.337	0.715
Sex	27.604	1	0.202	0.655
Age	33.774	1	0.247	0.621
MDS-UPDRS-III	14.138	1	0.104	0.749
PD cognitive classification*Cerebellum	96.336	2	0.353	0.704
Residuals	6413.839	47		

35. Linear Regression Model TUG dual-task by NBM volumes controlled for cognitive classification (interaction term), TUG single-task, sex, age, and MDS-UPDRS-III

	**TUG dual-task by NBM**				
Predictors	*Beta*	*CI (95%)*	*t-stats*	*p*	*p corr.*
Intercept (NC)	22.19	-7.97 – 52.35	1.48	0.15	
NBM	-56.11	-106.35 – -5.87	-2.25	**0.03**	0.6
SCI	-36.95	-84.09 – 10.18	-1.58	0.12	
MCI	-26.85	-58.20 – 4.49	-1.73	0.09	
Age	0.09	-0.14 – 0.32	0.78	0.44	
TUG single-task	1.14	0.62 – 1.66	4.45	**<0.001**	**0.001**
Sex	1.30	-0.91 – 3.52	1.18	0.24	
MDS-UPDRS III	0.00	-0.10 – 0.11	0.09	0.93	
SCI*NBM	81.12	-14.04 – 176.27	1.72	0.09	
MCI*NBM	57.91	-7.58 – 123.40	1.78	0.08	
Observations	55				
R^2 / R^2adjusted	0.543 / 0.452				
F(9,45) = 5.95, p = 1.915e-05, p corr. =4.3e-05					

ANOVA table

	SS	*df*	*F*	*p*	*p corr.*
Intercept (NC)	31.957	1	2.196	0.145	
NBM	73.639	1	5.059	**0.029**	0.6
PD cognitive classification	57.657	2	1.981	0.150	
Age	8.831	1	0.607	0.440	
TUG single-task	287.658	1	19.764	**0.000**	**0.001**
Sex	20.404	1	1.402	0.243	
MDS-UPDRS-III	0.117	1	0.008	0.929	
PD cognitive classification*NBM	63.854	2	2.194	0.123	
Residuals	654.969	45			

36. Linear Regression Model TUG difference by NBM volumes controlled for cognitive classification (interaction term), sex, age, and MDS-UPDRS-III

TUG difference by NBM

Predictors	*Beta*	*CI (95%)*	*t-stats*	*p*	*p corr.*
Intercept (NC)	-20.93	-50.48 – 8.62	-1.43	0.16	
NBM	55.59	5.80 – 105.38	2.25	**0.03**	0.6
SCI	34.59	-11.35 – 80.52	1.52	0.14	
MCI	26.85	-4.23 – 57.94	1.74	0.09	
Sex	-1.37	-3.56 – 0.82	-1.26	0.21	
Age	-0.12	-0.32 – 0.08	-1.18	0.24	
MDS-UPDRS III	-0.01	-0.11 – 0.09	-0.25	0.80	
SCI*NBM	-76.85	-169.92 – 16.22	-1.66	0.10	
MCI*NBM	-58.39	-123.31 – 6.54	-1.81	0.08	
Observations	55				
R^2 / R^2adjusted	0.219 / 0.084				
F(8,46) = 1.62, p = 0.15					

ANOVA table

	SS	*df*	*F*	*p*	*p corr.*
Intercept (NC)	29.124	1	2.032	0.161	
NBM	72.396	1	5.051	**0.029**	0.6
PD cognitive classification	55.142	2	1.924	0.158	
Sex	22.846	1	1.594	0.213	
Age	19.880	1	1.387	0.245	
MDS-UPDRS-III	0.932	1	0.065	0.800	
PD cognitive classification*NBM	61.727	2	2.153	0.128	
Residuals	659.302	46			

37. Linear Regression Model TUG dual-task by DLPFC volumes controlled for cognitive classification (interaction term), TUG single-task, sex, age, and MDS-UPDRS-III (interaction term)

	TUG dual-task by DLPFC				
Predictors	*Beta*	*CI (95%)*	*t-stats*	*p*	*p corr.*
Intercept (NC)	7.60	-25.24 – 40.44	0.47	0.64	
DLPFC	-24.50	-100.88 – 51.88	-0.65	0.52	
SCI	16.65	-35.25 – 68.54	0.65	0.52	
MCI	-10.69	-43.52 – 22.13	-0.66	0.51	
Age	0.15	-0.07 – 0.37	1.38	0.17	
TUG single-task	0.98	0.48 – 1.48	3.94	**<0.001**	**0.002**
Sex	2.28	0.11 – 4.46	2.12	**0.04**	0.6
MDS-UPDRS-III	-0.27	-0.48 – -0.05	-2.52	**0.02**	0.16
DLPFC * SCI	-73.81	-252.39 – 104.76	-0.83	0.41	
DLPFC * MCI	3.64	-91.24 – 98.53	0.08	0.94	
SCI * MDS-UPDRS-III	0.38	-0.09 – 0.84	1.64	0.11	
MCI * MDS-UPDRS-III	0.37	0.12 – 0.62	2.97	**<0.001**	**0.048**
Observations	55				
R^2 / R^2adjusted	0.605 / 0.504				
F(11,43) = 6.00, p = 8.03e-06, p corr. =2.0e-05					

ANOVA table

	SS	*df*	*F*	*p*	*p corr.*
Intercept (NC)	2.868	1	0.218	0.643	
DLPFC	5.505	1	0.418	0.521	
PD cognitive classification	18.285	2	0.695	0.505	
Age	25.181	1	1.914	0.174	
TUG single-task	203.927	1	15.498	**0.000**	**0.002**
Sex	59.230	1	4.501	**0.040**	0.4
MDS-UPDRS-III	83.268	1	6.328	**0.016**	0.15
PD cognitive classification*DLPFC	11.121	2	0.423	0.658	
PD cognitive classification*MDS-UPDRS-III	118.832	2	4.515	**0.017**	0.17
Residuals	565.814	43			

38. Linear Regression Model TUG difference by DLPFC volumes controlled for cognitive classification (interaction term), sex, age, and MDS-UPDRS-III

TUG difference by DLPFC

Predictors	*Beta*	*CI (95%)*	*t-stats*	*p*
Intercept (NC)	-7.02	-41.64 – 27.59	-0.41	0.68
DLPFC	30.18	-50.71 – 111.07	0.75	0.46
SCI	-15.02	-59.40 – 29.35	-0.68	0.50
MCI	-2.10	-34.98 – 30.77	-0.13	0.90
Sex	-1.73	-3.98 – 0.52	-1.55	0.13
Age	-0.08	-0.28 – 0.12	-0.80	0.43
MDS-UPDRS III	-0.01	-0.12 – 0.10	-0.20	0.85
SCI*DLPFC	38.88	-95.76 – 173.52	0.58	0.56
MCI*DLPFC	3.61	-95.59 – 102.81	0.07	0.94
Observations	55			
R^2 / R^2adjusted	0.189 / 0.048			
F(8,46) = 1.34, p = 0.25				

ANOVA table

	SS	*df*	*F*	*p*
Intercept (NC)	2.483	1	0.167	0.685
DLPFC	8.396	1	0.564	0.456
PD cognitive classification	7.314	2	0.246	0.783
Sex	35.698	1	2.398	0.128
Age	9.611	1	0.646	0.426
MDS-UPDRS-III	0.568	1	0.038	0.846
PD cognitive classification*DLPFC	5.491	2	0.184	0.832
Residuals	684.743	46		

39. Linear Regression Model TUG dual-task by hippocampus volumes controlled for cognitive classification (interaction term), TUG single-task, sex, age, and MDS-UPDRS-III (interaction term)

TUG dual-task by hippocampus

Predictors	*Beta*	*CI (95%)*	*t-stats*	*p*	*p corr.*
Intercept (NC)	16.74	-8.25 – 41.72	1.35	0.18	
Hippocampus	-30.00	-58.58 – -1.41	-2.12	**0.04**	1
SCI	-19.49	-47.74 – 8.76	-1.39	0.17	
MCI	-39.21	-65.40 – -13.01	-3.02	**<0.001**	0.17
Age	0.17	-0.06 – 0.39	1.49	0.14	

TUG single-task	0.97	0.48 – 1.45	3.98	**<0.001**	**0.002**
Sex	2.42	0.30 – 4.53	2.30	**0.03**	0.4
MDS-UPDRS-III	-0.28	-0.49 – -0.08	-2.76	**0.01**	0.16
SCI*hippocampus	27.52	-19.28 – 74.32	1.19	0.24	
MCI*hippocampus	46.94	7.18 – 86.70	2.38	**0.02**	0.8
SCI*MDS-UPDRS-III	0.20	-0.13 – 0.54	1.24	0.22	
MCI*MDS-UPDRS-III	0.42	0.17 – 0.66	3.45	**<0.001**	**0.04**
Observations	55				
R^2 / R^2 adjusted	0.636 / 0.543				
F(11,43) = 6.83, p = 1.739e-06, p corr. =5.4e-06					

ANOVA table

	SS	*df*	*F*	*p*	*p corr.*
Intercept (NC)	22.157	1	1.825	0.184	
Hippocampus	54.362	1	4.479	**0.040**	1
PD cognitive classification	110.959	2	4.571	**0.016**	0.2
Age	26.983	1	2.223	0.143	
TUG single-task	192.208	1	15.835	**0.000**	**0.002**
Sex	64.470	1	5.311	**0.026**	0.4
MDS-UPDRS-III	92.403	1	7.612	**0.008**	0.16
PD cognitive classification*Hippocampus	69.816	2	2.876	0.067	
PD cognitive classification*MDS-UPDRS-III	152.679	2	6.289	**0.004**	0.1
Residuals	521.956	43			

40. Linear Regression Model TUG difference by hippocampus controlled for cognitive classification (interaction term), sex, age, and MDS-UPDRS-III

	**TUG difference by Hippocampus**			
Predictors	*Beta*	*CI (95%)*	*t-stats*	*p*
Intercept (NC)	-15.24	-42.10 – 11.63	-1.14	0.26
Hippocampus	30.86	-0.49 – 62.21	1.98	0.05
SCI	10.82	-19.32 – 40.96	0.72	0.47
MCI	19.98	-5.73 – 45.69	1.56	0.12
Sex	-1.58	-3.81 – 0.64	-1.43	0.16
Age	-0.09	-0.29 – 0.12	-0.84	0.40
MDS-UPDRS III	-0.01	-0.11 – 0.09	-0.20	0.84
SCI*Hippocampus	-22.22	-71.96 – 27.53	-0.90	0.37

MCI*Hippocampus	-35.07	-77.32 – 7.19	-1.67	0.10
Observations	55			
R^2 / R^2 adjusted	0.200 / 0.061			
F(8,46) = 1.44, p = 0.21				

ANOVA table

	SS	*df*	*F*	*p*
Intercept (NC)	19.150	1	1.304	0.259
Hippocampus	57.682	1	3.927	0.054
PD cognitive classification	36.052	2	1.227	0.303
Sex	30.122	1	2.050	0.159
Age	10.425	1	0.710	0.404
MDS-UPDRS-III	0.613	1	0.042	0.839
PD cognitive classification*Hippocampus	41.693	2	1.419	0.252
Residuals	675.763	46		

41. Linear Regression Model TUG dual-task by cerebellum volumes controlled for cognitive classification (interaction term), TUG single-task, sex, age, and MDS-UPDRS-III (interaction term)

TUG dual-task by cerebellum

Predictors	*Beta*	*CI (95%)*	*t-stats*	*p*	*p corr.*
Intercept (NC)	6.70	-27.49 – 40.90	0.40	0.69	
Cerebellum	-12.50	-72.24 – 47.25	-0.42	0.68	
SCI	-40.33	-78.30 – -2.36	-2.14	**0.04**	0.8
MCI	1.93	-32.19 – 36.05	0.11	0.91	
Age	0.11	-0.09 – 0.31	1.10	0.28	
TUG single-task	1.19	0.72 – 1.65	5.12	**<0.001**	**0.0003**
Sex	2.11	0.09 – 4.13	2.10	**0.04**	0.4
MDS-UPDRS-III	-0.28	-0.48 – -0.08	-2.84	**0.01**	0.16
SCI*Cerebellum	82.74	2.72 – 162.75	2.09	**0.04**	1
MCI*Cerebellum	-25.84	-96.68 – 45.00	-0.74	0.47	
SCI*MDS-UPDRS-III	0.10	-0.22 – 0.42	0.61	0.54	
MCI*MDS-UPDRS-III	0.36	0.13 – 0.59	3.15	**<0.001**	**0.04**
Observations	55				
R^2 / R^2 adjusted	0.664 / 0.578				
F(11,43) = 7.72, p = 3.741e-07, p corr. =1.4e-04					

ANOVA table

	SS	***df***	***F***	***p***	***p corr.***
Intercept (NC)	1.752	1	0.156	0.695	
Cerebellum	1.994	1	0.178	0.675	
PD cognitive classification	86.772	2	3.871	**0.028**	0.2
Age	13.459	1	1.201	0.279	
TUG single-task	293.714	1	26.208	**0.000**	**0.0003**
Sex	49.525	1	4.419	**0.041**	0.4
MDS-UPDRS-III	90.436	1	8.069	**0.007**	0.16
PD cognitive classification*Cerebellum	115.389	2	5.148	**0.010**	0.4
PD cognitive classification*MDS-UPDRS-III	132.922	2	5.930	**0.005**	0.1
Residuals	481.910	43			

42. Linear Regression Model TUG difference by cerebellum volumes controlled for cognitive classification (interaction term), sex, age, and MDS-UPDRS-III

	**TUG difference by cerebellum**			
Predictors	*Beta*	*CI (95%)*	*t-stats*	*p*
Intercept (NC)	4.99	-31.87 – 41.85	0.27	0.79
cerebellum	-0.29	-65.01 – 64.44	-0.01	0.99
SCI	23.48	-16.60 – 63.56	1.18	0.24
MCI	-22.63	-57.28 – 12.03	-1.31	0.20
Sex	-1.57	-3.73 – 0.59	-1.47	0.15
Age	-0.12	-0.31 – 0.07	-1.25	0.22
MDS-UPDRS III	0.02	-0.08 – 0.12	0.44	0.66
SCI*cerebellum	-53.76	-138.92 – 31.39	-1.27	0.21
MCI*cerebellum	47.77	-28.45 – 124.00	1.26	0.21
Observations	55			
R^2 / R^2 adjusted	0.259 / 0.130			
$F(8,46) = 2.01$, $p = 0.07$				

ANOVA table

	SS	***df***	***F***	***p***	***p corr.***
Intercept (NC)	1.010	1	0.074	0.786	
Cerebellum	0.001	1	0.000	0.993	
PD cognitive classification	103.068	2	3.786	**0.030**	0.2
Sex	29.250	1	2.149	0.149	
Age	21.319	1	1.566	0.217	
MDS-UPDRS-III	2.674	1	0.196	0.660	
PD cognitive classification*Cerebellum	110.078	2	4.043	**0.024**	0.5
Residuals	626.176	46			
